# Functional Transcriptome Analysis in ARSACS KO Cell Model Reveals a Role of Sacsin in Autophagy

**DOI:** 10.1038/s41598-019-48047-x

**Published:** 2019-08-15

**Authors:** Federica Morani, Stefano Doccini, Roberto Sirica, Marta Paterno, Francesco Pezzini, Ivana Ricca, Alessandro Simonati, Massimo Delledonne, Filippo Maria Santorelli

**Affiliations:** 1 Molecular Medicine for Neurodegenerative and Neuromuscular Diseases Unit, IRCCS Stella Maris Foundation, Pisa, 56128 Italy; 2 Department of Biotechnology, University of Verona, Verona, 37134 Italy; 3 Neurology (Neuropathology and Child Neurology), Department of Neuroscience, Biomedicine and Movement, University of Verona, Verona, 37134 Italy

**Keywords:** Spinocerebellar ataxia, Genetics of the nervous system

## Abstract

Autosomal recessive spastic ataxia of Charlevoix-Saguenay (ARSACS) is a rare early-onset neurological disease caused by mutations in *SACS*, which encodes sacsin. The complex architecture of sacsin suggests that it could be a key player in cellular protein quality control system. Molecular chaperones that operate in protein folding/unfolding and assembly/disassembly patterns have been described as essential modulators of selectivity during the autophagy process. We performed RNA-sequencing analysis to generate a whole-genome molecular signature profile of sacsin knockout cells. Using data analysis of biological processes significantly disrupted due to loss of sacsin, we confirmed the presence of decreased mitochondrial function associated with increased oxidative stress, and also provided a demonstration of a defective autophagic pathway in sacsin-depleted cells. Western blotting assays revealed decreased expression of LC3 and increased levels of p62 even after treatment with the lysosomal inhibitor bafilomycin A1, indicating impairment of the autophagic flux. Moreover, we found reduced co-immunolocalization of the autophagosome marker LC3 with lysosomal and mitochondrial markers suggesting fusion inhibition of autophagic compartments and subsequent failed cargo degradation, in particular failed degradation of damaged mitochondria. Pharmacological up-regulation of autophagy restored correct autophagic flux in sacsin knockout cells. These results corroborate the hypothesis that sacsin may play a role in autophagy. Chemical manipulation of this pathway might represent a new target to alleviate clinical and pathological symptoms, delaying the processes of neurodegeneration in ARSACS.

## Introduction

Spastic ataxia of Charlevoix-Saguenay (ARSACS) is a childhood-onset neurological disease characterized by cerebellar ataxia, lower limb pyramidal tract signs, and axonal-demyelinating sensorimotor peripheral neuropathy^1–4^. Patients with ARSACS usually develop signs of the disease before the age of 5 years, although a small proportion of cases become symptomatic in adulthood^5^. ARSACS results from bi-allelic mutations in *SACS*^6,7^ and it shows variable phenotypic expression. At the milder end of the spectrum, clinical features include mild intellectual disability, retinal disturbance and autonomic symptoms^4,8,9^. To date, over 170 *SACS* mutations have been identified worldwide, mostly causing loss of function of the 520 kDa multidomain gene product sacsin^7,10^. The domain composition of sacsin has, in part, been clarified. From the N- to the C-terminus, sacsin structure consists of: an ubiquitin-like domain that binds to the proteasome; three large sacsin repeating regions that share an Hsp90-like chaperone function; an XPCB domain that interacts with the ubiquitin ligase Ube3A; a DnaJ domain binding Hsc70 (member of the Hsp70 chaperone family); and a higher eukaryote and prokaryote nucleotide-binding domain that mediates sacsin dimerization and binds to nucleotides or their analogs^11–17^. The nature and architecture of these modules suggest that sacsin is involved in protein quality control; this would be consistent with the role that other molecular chaperones are increasingly recognized to play in neurodegeneration, specifically as key mediators of: protein homeostasis (proteostasis) in the ubiquitin-proteasome system; endoplasmic reticulum-associated degradation; and different autophagic pathways, including chaperone-mediated-, micro-, and macro-autophagy, and organelle-specific processes. Using patient-derived cell lines, cell models (e.g., SH-SY5Y, Cos-7), primary neuronal cultures from *Sacs*^−/−^ mice, and murine brain slice cultures, it has been demonstrated that sacsin localizes to mitochondria and that sacsin depletion leads to alterations in mitochondrial bioenergetics and dynamics, resulting in neuronal cell death^18,19^. Study of mitochondrial networks in ARSACS patient cells^20–22^ suggests that sacsin promotes mitophagy following mitochondrial damage. However, the link(s) between the putative chaperone function of sacsin and its role in mitochondrial dynamics and bioenergetics remain unclear. Importantly from the perspective of the present study, it has been demonstrated that molecular chaperones, implicated in protein folding/unfolding and assembly/disassembly, are key modulators of selectivity during the autophagy process^23^. Hsc70 has been associated with endosomal microautophagy^24^ and chaperone-assisted selective autophagy (CASA), a sub-type of macroautophagy which allow the degradation of protein aggregates or damaged organelles^23^.

We hypothesized that sacsin acts in the autophagic machinery, mediating CASA, and that its loss may lead to impaired autophagic flux, and, in turn, impaired mitochondrial degradation. Herein we show that mTOR-mediated autophagy is differentially regulated in sacsin-depleted cells (KO cells), and that pharmacological induction of autophagy restores a correct flux. Clarification of these mechanisms, in addition to improving our biological understanding of how sacsin operates in disease conditions, might also suggest that chemical manipulation of the autophagic pathways is a valid new avenue to pursue in the pharmacological approach to ARSACS.

## Results

### Characterization of sacsin KO cell line

To better investigate the role of sacsin in ARSACS, we used the CRISPR/Cas9 editing technology to generate a sacsin KO cell line using SH-SY5Y neuroblastoma cells (Supplementary Fig. S1). Correct gene editing was verified by Sanger sequencing in all clones. Among 18 clones with indel mutations in the *SACS* gene, we selected for further studies, one clone harboring a 100 bp insertion (SH-SY5Y_clone 1B in exon 2, resulting in a premature stop codon (Supplementary Fig. S1A). Western blotting showed that cells derived from this clone had undetectable levels of sacsin, as expected (Supplementary Fig. S1B).

**Figure 1:**
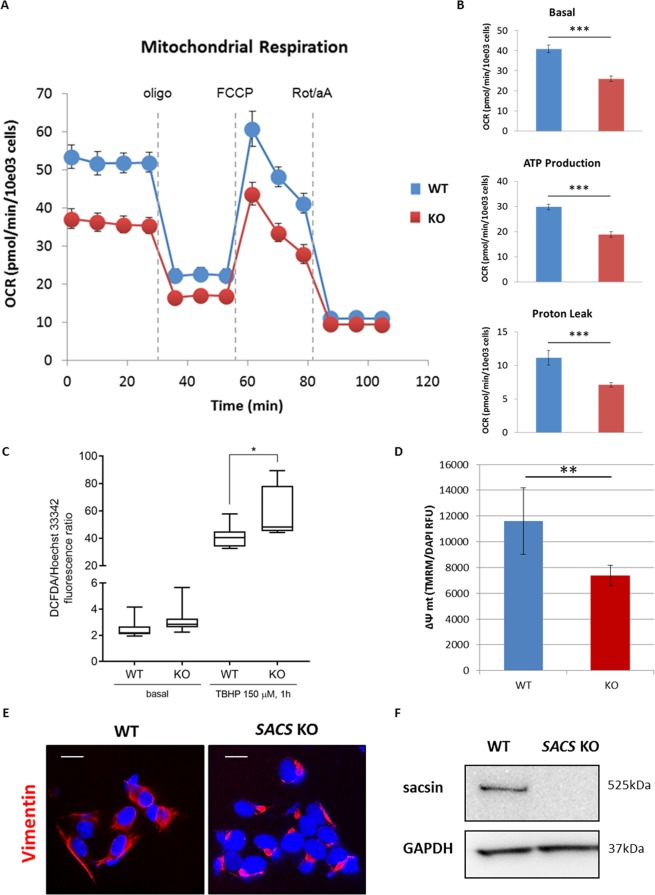
Mitochondrial bioenergetic function reduction, ROS levels increase, mitochondrial membrane potential impairment and abnormal intermediate filament network in sacsin KO cells. Measurement of OCR (**A**), basal respiration, ATP production and proton leak (**B**) in WT and KO cells using the Agilent Seahorse XF Cell Mito Stress Test. The assay was performed under basal conditions and after addition of olygomycin (2 µM), carbonyl cyanide 4-trifluoromethoxyphenylhydrazone (FCCP) (1.5 µM) and rotenone plus antimycin A (1 µM). Comparison between WT and sacsin KO cells showed impaired mitochondrial function (OCR = oxygen consumption rate; oligo = oligomycin; Rot = rotenone; aA = antimycin A). (**C**) Fluorimetric detection of intracellular ROS in WT and sacsin KO cells in basal condition (only addition of 2′,7′–dichlorofluorescin diacetate (DCFDA), 25 µM) and after tert-butyl hydroperoxide (TBHP) treatment (150 µM) using DCFDA assay kit showed a significant increase in intracellular ROS levels in KO cells after oxidative stress induction. Hoechst 33342 was used to normalize cell number. (**D**) Cells were loaded with the fluorescent cationic probe tetramethylrhodamine methyl ester (TMRM). TMRM, whose fluorescence intensity was measured using the Spectramax iD3 microplate reader, showed a significantly reduced Δψm in KO cells. Δψm was normalized by DAPI fluorescence, as a function of number of cells. (RFU = relative fluorescence units; Δψm = mitochondrial membrane potential). *p < 0.05; **p < 0.01; and ***p < 0.001. (**E**) Representative images of vimentin network (in red) in WT and sacsin KO cells showed a collapsed intermediate filament network in cells lacking sacsin. DAPI (in blue) was used as nuclear stain. Scale bar = 10 µm. (**F**) Western blotting showed undetectable sacsin levels in KO cell line. GAPDH was used as a loading control. Full-length blots are presented in the Supplementary Information 1.

We demonstrated that KO cells are a valid model in ARSACS. Consistent with data presented by us^21^ and others^20^ on the role of sacsin in mitochondrial bioenergetics, we observed that sacsin KO cells showed a decreased oxygen consumption rate (OCR) both before and after the addition of respiratory chain inhibitors and uncouplers (Fig. 1A). Microrespirometry revealed a reduction in basal respiration, ATP production and proton leak levels in KO cells (Fig. 1B). In the presence of 2′,7′–dichlorofluorescin diacetate (DCFDA), a marker of cellular reactive oxygen species (ROS)-mediated DNA damage, we detected no significant changes in free radical production in KO cells (Fig. 1C) under basal conditions. Conversely, exposure of cells to tert-butyl hydrogen peroxide (TBHP) caused a significant increase of fluorescence in cells where sacsin was absent (Fig. 1C), a finding consistent with the increased oxidative stress shown in cultured ARSACS skin fibroblasts and in cells where sacsin had been transiently shut down^20,21^. Finally, we evaluated Δψm loading our cell model with the fluorescent dye tetramethylrodamine methyl (TMRM), a cationic probe with minimal phototoxicity and low photobleaching that accumulates in polarized mitochondria and is released when the membrane potential decreases. Sacsin KO cells showed a significantly lower Δψm compared to a wild-type (WT) CRISPR control line (Fig. 1D), confirming the presence of impaired mitochondrial function. In addition, we observed altered perinuclear vimentin collapsed network in in KO SH-SY5Y cells (Fig. 1E), an indicator of disorganized intermediate filaments as already seen in KO HEK-293T^22^. On the whole, our data support sacsin KO SH-SY5Y cell line as a viable model of ARSACS.

### Comparative analysis of transcriptome profiles in WT and sacsin KO cells

RNA-seq transcriptome analysis, a challenging but precise transcriptome-profiling method, was used to generate a whole-genome molecular signature profile of sacsin KO SH-SY5Y cells as compared to WT SH-SY5Y cells expressing sacsin, in an attempt to shed light on the consequences of this genetic alteration^25^. Differentially expressed genes (DEGs) were, in turn, examined querying bioinformatics tools to recognize relevant involved biological functions and molecular pathways.

**Figure 2:**
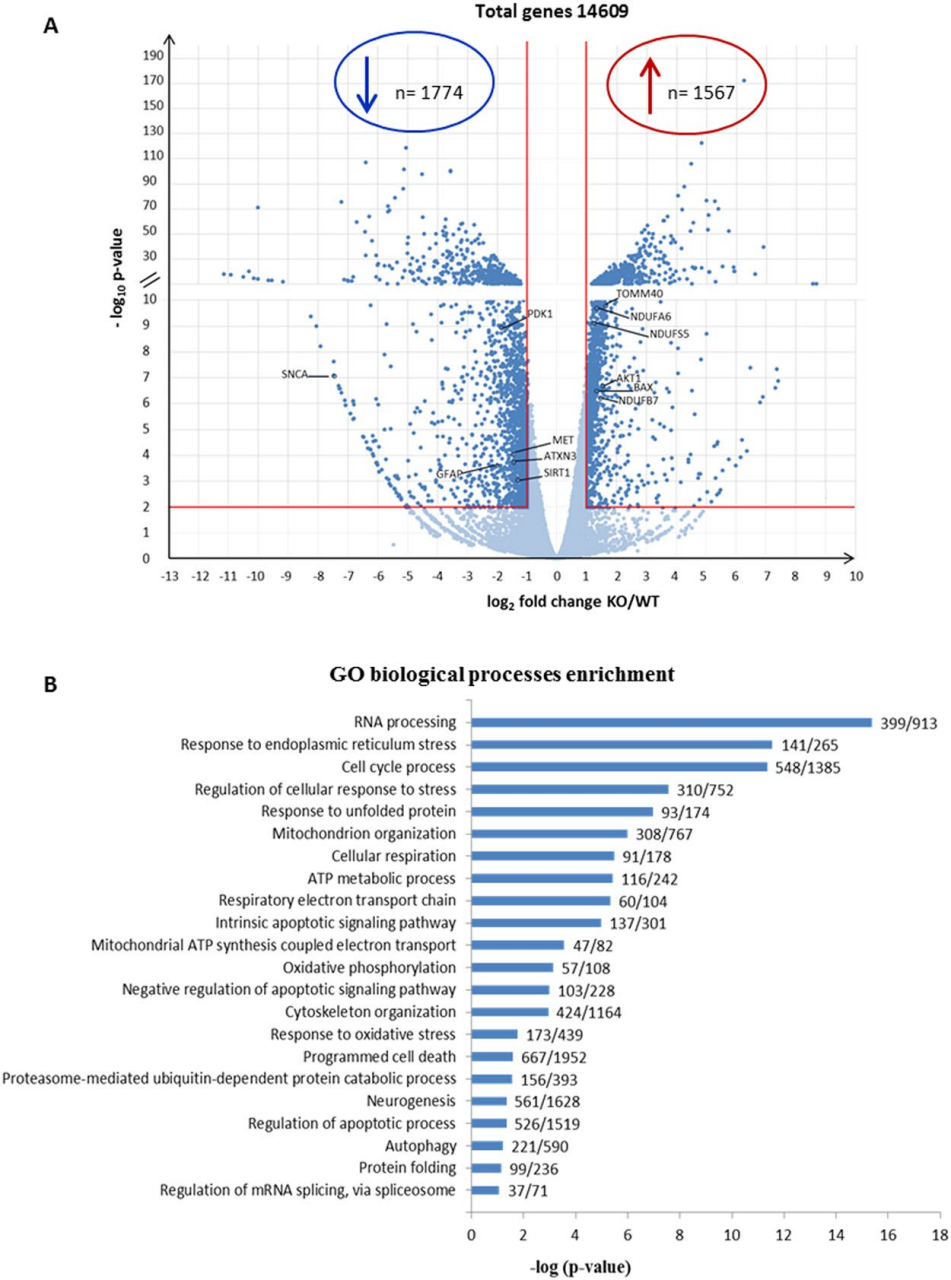
Altered expression of genes identified through RNA-seq analyses comparing sacsin KO cells and WT control cells. (**A**) Volcano plot of transcriptome analyses of KO cells compared with WT cells. Names are indicated for some relevant genes. p value < 0.01. (**B**) Significantly enriched biological processes illustrated by transcriptomic data.

The transcriptome profiles revealed 3341/14609 differentially expressed transcripts in KO cells (Fig. 2A) of which 1567 were up-regulated and 1774 down-regulated, with a fold change >2 and statistical significance set at p < 0.01. Categorization of DEGs identified in KO cells through the bioinformatic suites ToppFun and iPathwayGuide identified *RNA processing, mitochondrion organization, cellular respiration* and *oxidative phosphorylation* (Fig. 2B) as the most meaningful *Molecular and cellular functions* categories. In addition, *Programmed cell death, Autophagy* and *Protein folding* were among the three highest-ranking categories related to *Physiological system development and function*, suggesting an alteration of neurobiological processes related to maintenance of cell homeostasis. In particular, a specific heat map of the autophagy process revealed 221 DEGs: 106 up-regulated (in Supplementary Fig. S2A) and 115 down-regulated genes (in Supplementary Fig. S2B), while the heat map of oxidative phosphorylation showed 71 DEGs (Supplementary Fig. S3). Interaction network linking *Apoptosis of central nervous system cells* from *Disease and functions* category with 55 DEGs showed an activation z-score = 1.555 indicating the up-regulation of cell death in KO cells (Supplementary Fig. S4).

### Ubiquitin-proteasome quality control system is not altered in KO cells

**Figure 3:**
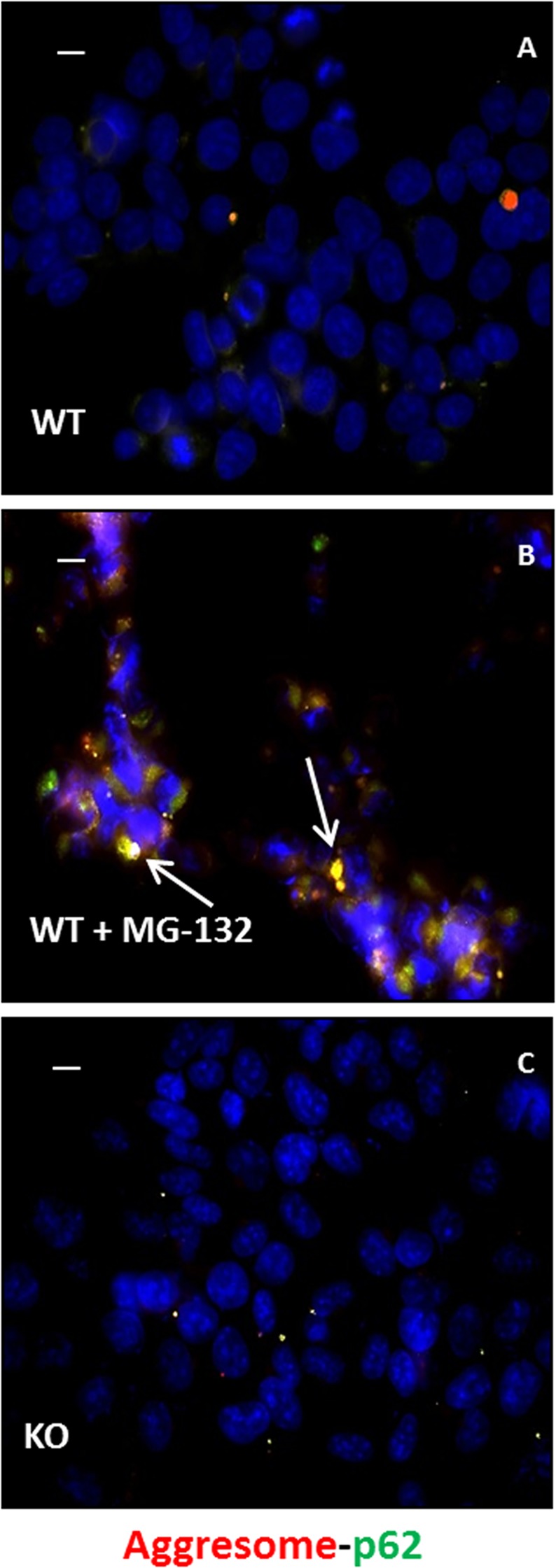
Aggresome staining in WT and sacsin KO cells. Representative images of p62 (in green) and aggresomes (in red) in WT and sacsin KO cells under normal conditions (**A,C**) or in WT cells after MG-132 treatment (**B**). Hoechst 33342 (in blue) was used as nuclear stain. Colocalization between aggresomes and p62 was absent in sacsin KO cells. Colocalization (in yellow) is indicated by arrows. Scale bar = 10 µm.

To detect possible alteration of the autophagy system or the ubiquitin-proteasome quality control system in cells lacking sacsin, we determined the presence of aggregate structures formed in the cytoplasm of sacsin KO cells. This was done using the ProteoStat® Aggresome Detection kit (Enzo LifeSciences, Lausanne, Switzerland). In line with previous observations in ARSACS primary fibroblasts^22^, no aggresome-like inclusions were found in our KO and WT cell model, unlike what was observed when aggregation was induced in WT cells by treatment with the proteasome inhibitor MG-132 (Fig. 3). We also excluded proteasome impairment in cells lacking sacsin. Indeed, using p62 co-immunolocalization to characterize the autophagic nature of the aggresomes, we observed proper working of the ubiquitin-proteasome system in KO cells under basal conditions (Fig. 3C).

### mTOR-dependent autophagy induction by FCCP treatment is impaired in sacsin KO cells

**Figure 4:**
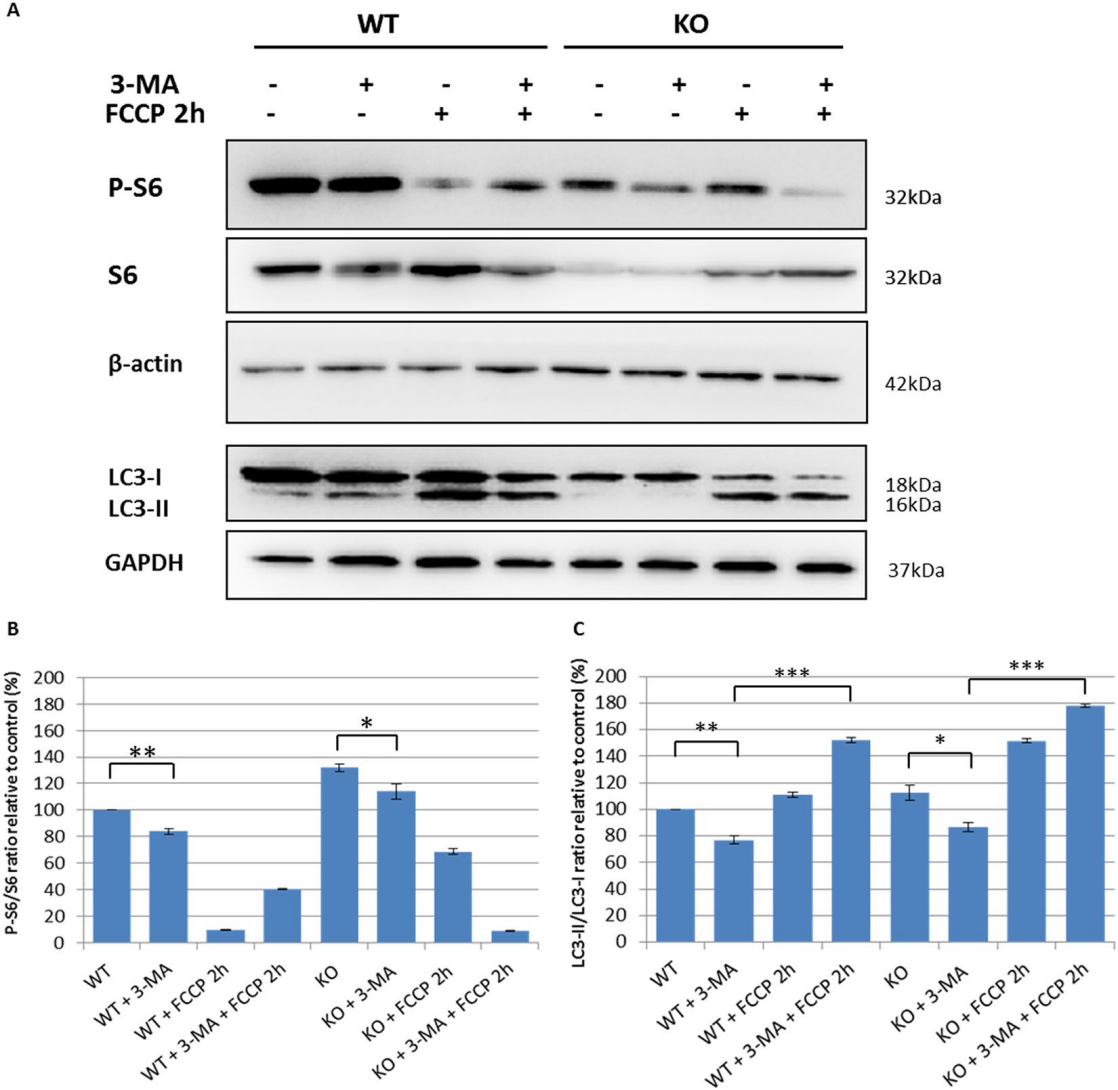
m-TOR dependent autophagy in WT and sacsin KO cell line. (**A**) Western blotting analysis of phospho-S6 (P-S6) and total S6 (S6) and LC3 autophagy markers in WT and sacsin KO cells treated for 2 h with the mitochondrial uncoupler carbonyl cyanide 4-trifluoromethoxyphenylhydrazone (FCCP 20 µM) in the absence or the presence of the PI3K inhibitor 3-methyladenine (3-MA; 10 mM). Full-length blots are presented in the Supplementary Information 2. The densitometry ratios of P-S6/S6 normalized versus β-actin (**B**) and of LC3-II/LC3-I normalized versus GAPDH (**C**) are reported, and they show m-TOR dependent regulation of autophagy in both cell lines. The data shown in this figure were reproduced independently three times. ns, not statistically significant; *p < 0.05; **p < 0.01; and ***p < 0.001.

**Figure 5:**
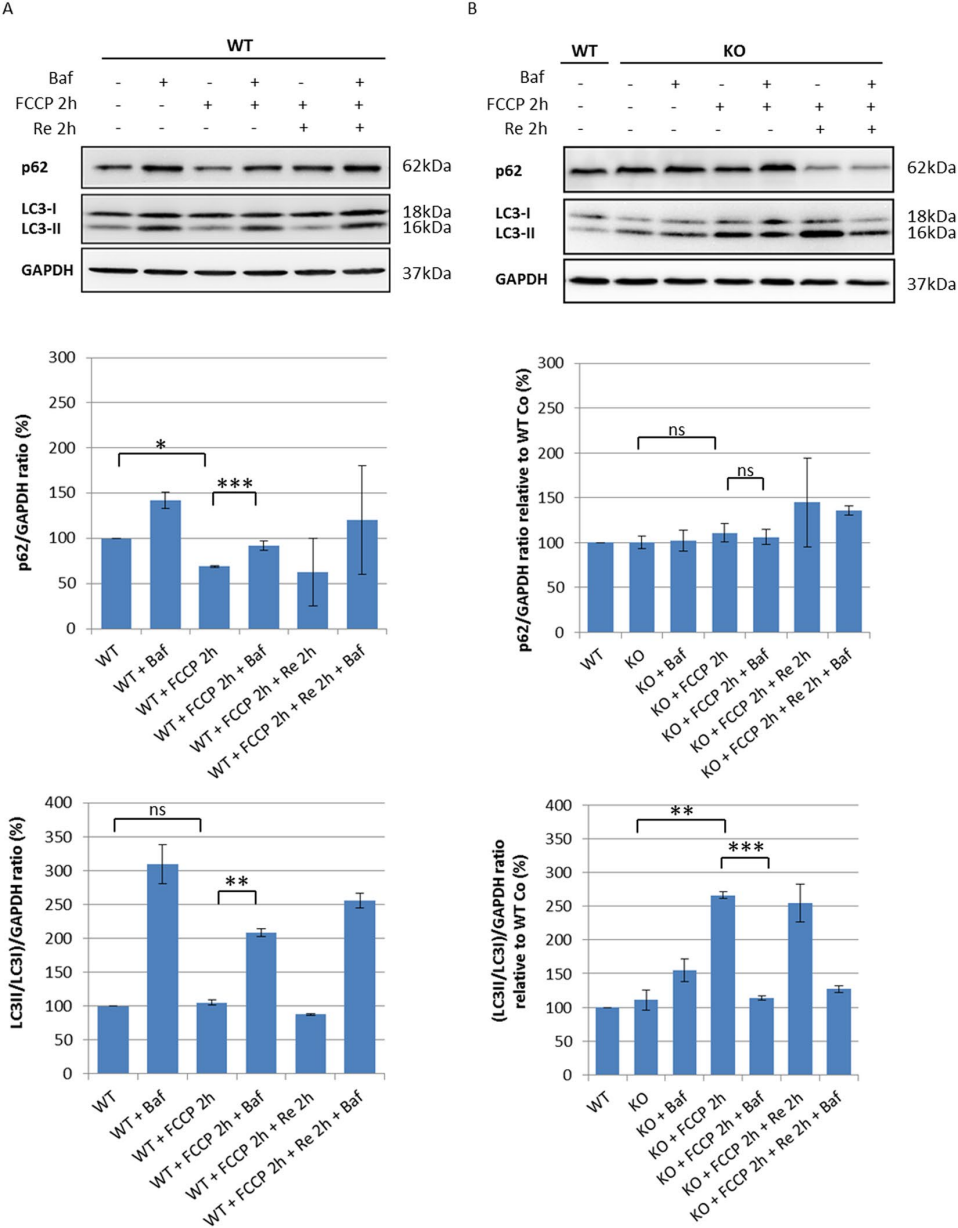
The autophagic flux is impaired in sacsin KO cells. (**A**) Western blotting analysis of LC3 and p62 autophagy markers in WT cells treated for 2 h with the mitochondrial uncoupler FCCP (20 µM) in the absence or the presence of the lysosomal inhibitor bafilomycin A1 (200 nM). The densitometry ratios of LC3-II/LC3-I and of p62 normalized versus GAPDH are reported, and they show correct autophagic flux induction by carbonyl cyanide 4-trifluoromethoxyphenylhydrazone (FCCP) treatment. (**B**) Western blotting analysis showing LC3 and p62 levels in sacsin KO cells treated for 2 h with the uncoupler FCCP (20 µM) in the absence or the presence of bafilomycin A1 (200 nM). The densitometry ratios of LC3-II/LC3-I and p62 normalized vs GAPDH showed the failure of LC3-II to increase in the presence of bafilomycin, even after normal medium recovery, and p62 accumulation, these findings together indicate a defective autophagy process in KO cells.Data shown in this figure were reproduced independently three times. ns, not statistically significant; *p < 0.05; **p < 0.01; and ***p < 0.001. (Baf = bafilomycin A1; Re = normal medium recovery). Full-length blots are presented in the Supplementary Information 3.

To establish whether regulation of autophagy in our cell models of ARSACS occurred via an mTOR dependent or independent pathway, we pre-treated cells with 3-methyladenine (3-MA), a phosphoinositide 3-kinase (PI3K) inhibitor, 3 h before treatment with carbonyl cyanide 4-trifluoromethoxyphenylhydrazone (FCCP), and analyzed expression levels of phospho-S6, an effector of the mTOR pathway, and total S6. 3-MA is an autophagy inhibitor that blocks class I PI3K persistently, whereas its suppressive effect on class III PI3K is transient^26^. As shown in Fig. 4, the effectiveness of 3-MA was confirmed by the reduction of S6 protein phosphorylation, a surrogate marker of mTOR activity, in both WT and sacsin KO cells, suggesting correct mTOR regulation of autophagy in ARSACS (Fig. 4A,B). Instead, an inhibitory effect of 3-MA on class III PI3K proved insufficient to completely abolish the induction of autophagy by FCCP treatment in both cell lines (Fig. 4C). Thereafter, to investigate the stages in the autophagy process in greater depth, we analyzed LC3 and p62 protein levels in basal condition and after mitophagy induction by the mitochondrial uncoupler FCCP in sacsin KO cells. Whilst WT cells showed normal activation of autophagy by FCCP, with reduced levels of p62^27^, KO cells showed a massive increase of LC3-II signal without changes in p62 signal, consistent with an aberrant accumulation of autophagic vesicles (Fig. 5A,B). Failure of LC3-II to increase in the presence of reagents that inhibit acidification within lysosomes or inhibit autophagosome–lysosome fusion indicates a defect or delay in the autophagy process^28^. We used bafilomycin A1, a specific lysosomal inhibitor of vacuolar-type H + -ATPase, which blocks degradation of autophagosome content, including LC3, to assess whether sacsin depletion could inhibit autophagic flux. As shown in Fig. 5, bafilomycin A1 treatment led to a marked increase in LC3-II bands in WT but not in KO cells. Sacsin KO cells also showed unchanged levels of p62 compared with WT cells, confirming a defect in the turnover of poly-ubiquitinated protein aggregates. Combined, these data showed that autophagic flux is impaired when sacsin is knocked out.

**Figure 6:**
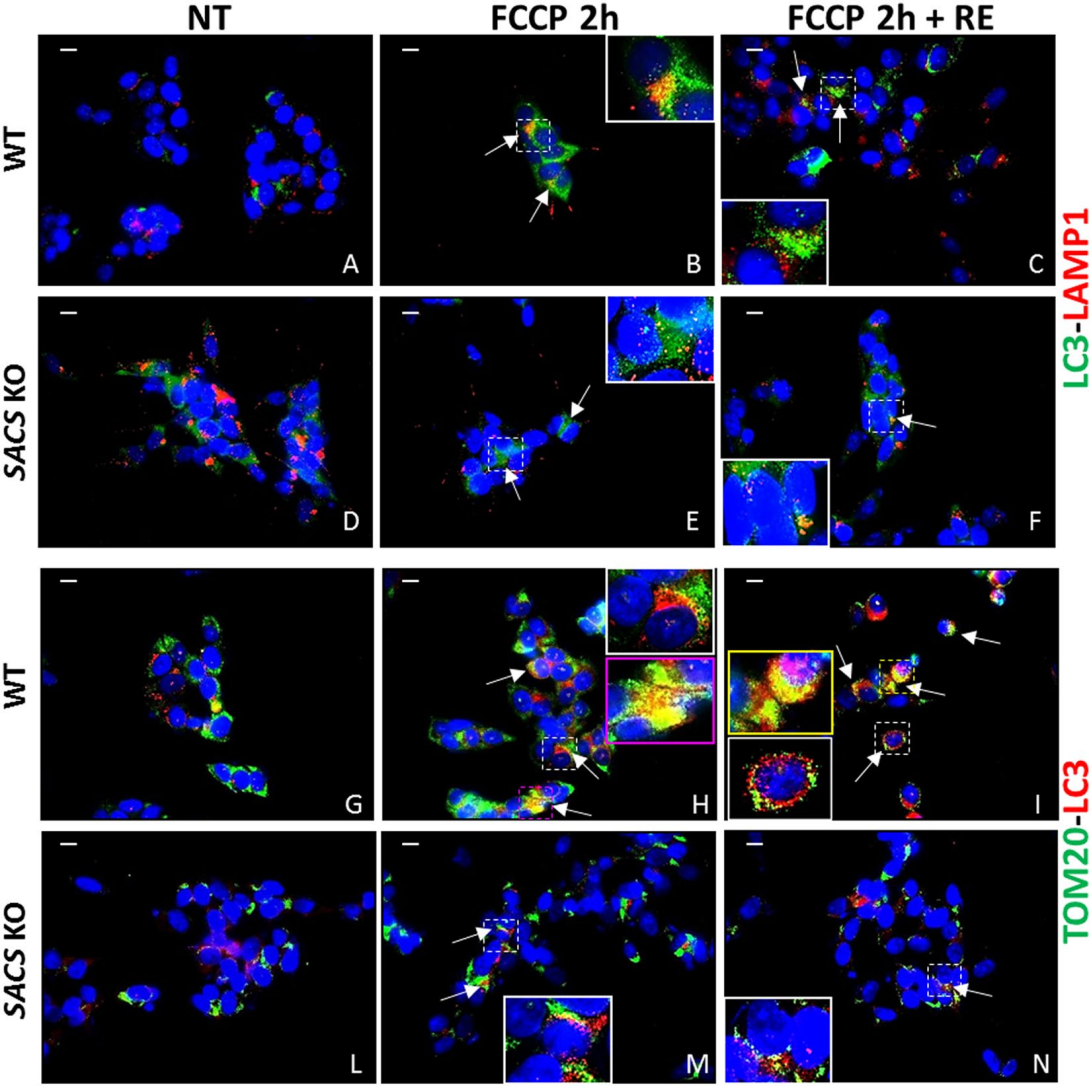
Mitophagy is impaired in sacsin KO cells. (Panels A–F) Autophagosome-lysosome fusion is disrupted in sacsin KO cells. Immunofluorescence images of LC3 (in green) and LAMP1 (in red) in WT and sacsin KO cells under normal conditions (**A,D**), FCCP treatment (**B,E**) and FCCP treatment followed by medium recovery (**C,F**). DAPI (in blue) was used as nuclear stain. Fusion between autophagosomes and lysosomes was reduced in sacsin KO cells even after medium recovery. Colocalization of LC3 with LAMP1 (in yellow) is indicated by arrows. (Panels G–N) Mitophagy activation is reduced in KO cells. Immunofluorescence images of the mitochondrial marker TOM20 (in green) and LC3 (in red) in WT and sacsin KO cells under normal conditions (**G,L**) FCCP treatment (**H,M**) and FCCP treatment followed by medium recovery (**I,N**). DAPI (in blue) was used as nuclear stain. Fusion between mitochondria and autophagosomes was reduced in sacsin KO cells even after medium recovery. Colocalization of LC3 with TOM20 (in yellow) is indicated by arrows. Scale bar = 10 µm. (Re = normal medium recovery).

**Figure 7:**
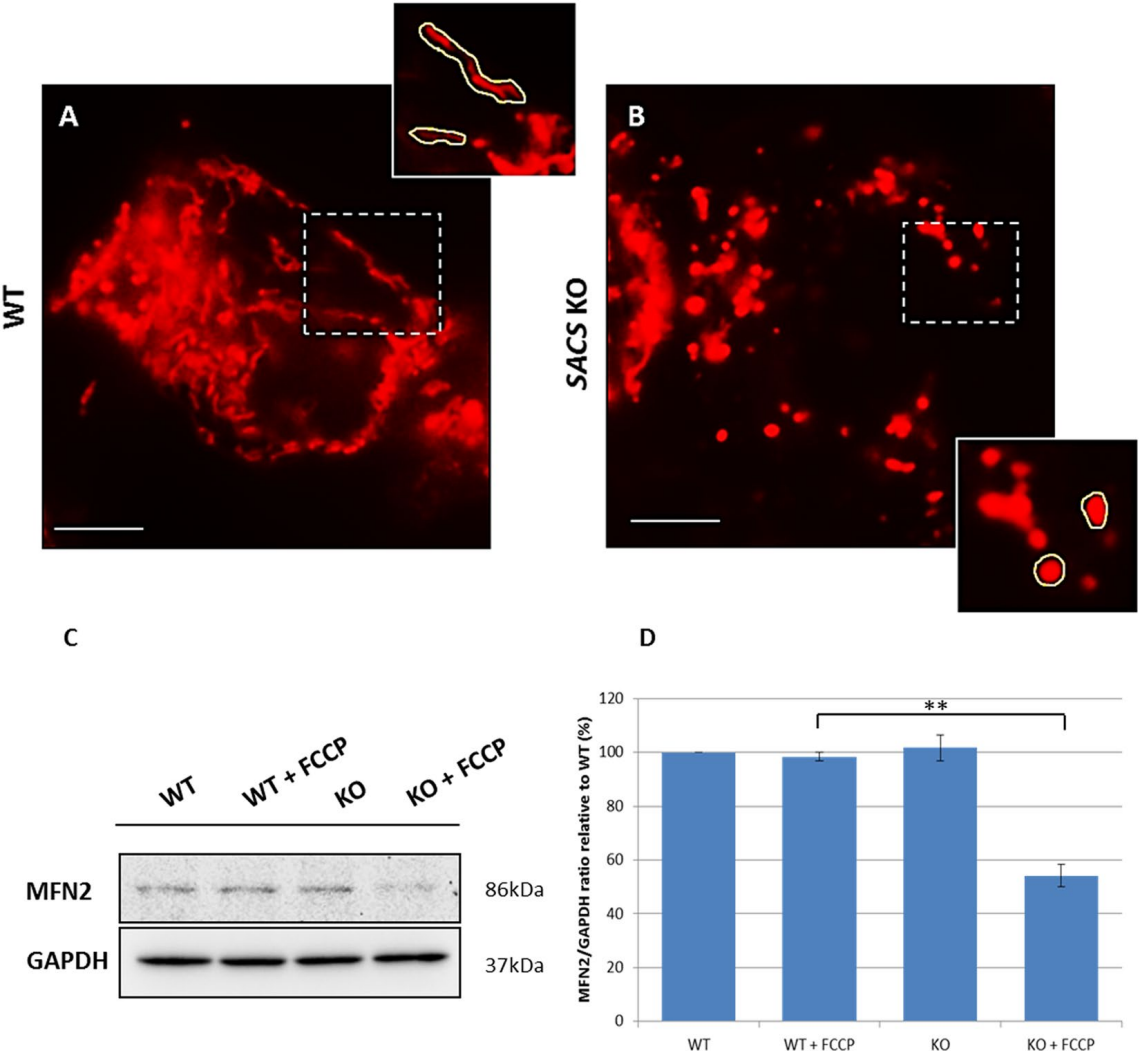
Mitochondrial pattern is disrupted in sacsin KO cells. (Panels A,B) Representative images showing WT and KO cells that were stained with MitoTracker (in red) under normal conditions. Mitochondria appeared more fragmented in KO cells as opposed to WT cells as highligthed by yellow border in the magnification inserts. Scale bar = 10 µm. (**C**) Western blotting of MFN2 in WT and KO cells in the presence or absence of the mitochondrial uncoupler FCCP (20 µM, 2 h). Full-length blots are presented in the Supplementary Information 4. (**D**) Densitometry of protein bands normalized versus GAPDH showed a significant reduced steady-state levels of MFN2 in KO cells after FCCP treatment. **p < 0.01.

Mitophagy, the selective removal of dysfunctional mitochondria via autophagy, is an organelle-specific pathway that accompanies the more general macroautophagy process (often referred to simply as autophagy) that is frequently linked to neurodegenerative conditions^29^. We assayed mitophagy by performing immunofluorescence assays under normal conditions, FCCP treatment, and FCCP treatment followed by medium recovery (Fig. 6). Double immunolabeling with LC3 and LAMP1 (Fig. 6A–F) during FCCP treatment showed impaired autophagosome formation and reduced fusion with the lysosomes even after medium recovery in sacsin KO cells (Fig. 6E,F) compared with WT cells (Fig. 6B,C). In addition, there was abundant colocalization of LC3 puncta with TOM20, a translocase of the outer mitochondrial membrane in WT (Fig. 6H) but not in KO cells (Fig. 6M), even after normal medium recovery (Fig. 6N), suggesting a defective mitophagy activation process when sacsin is absent. Moreover we analyzed mitochondrial network using the dye MitoTracker Red observing a more fragmented pattern in KO cells (Fig. 7A) compared to WT line (Fig. 7B). In keeping with these data, we also observed low mitofusin2 (MFN2) expression after FCCP-induced fragmentation confirming an impaired mitochondrial dynamic in KO cells (Fig. 7C,D).

**Figure 8:**
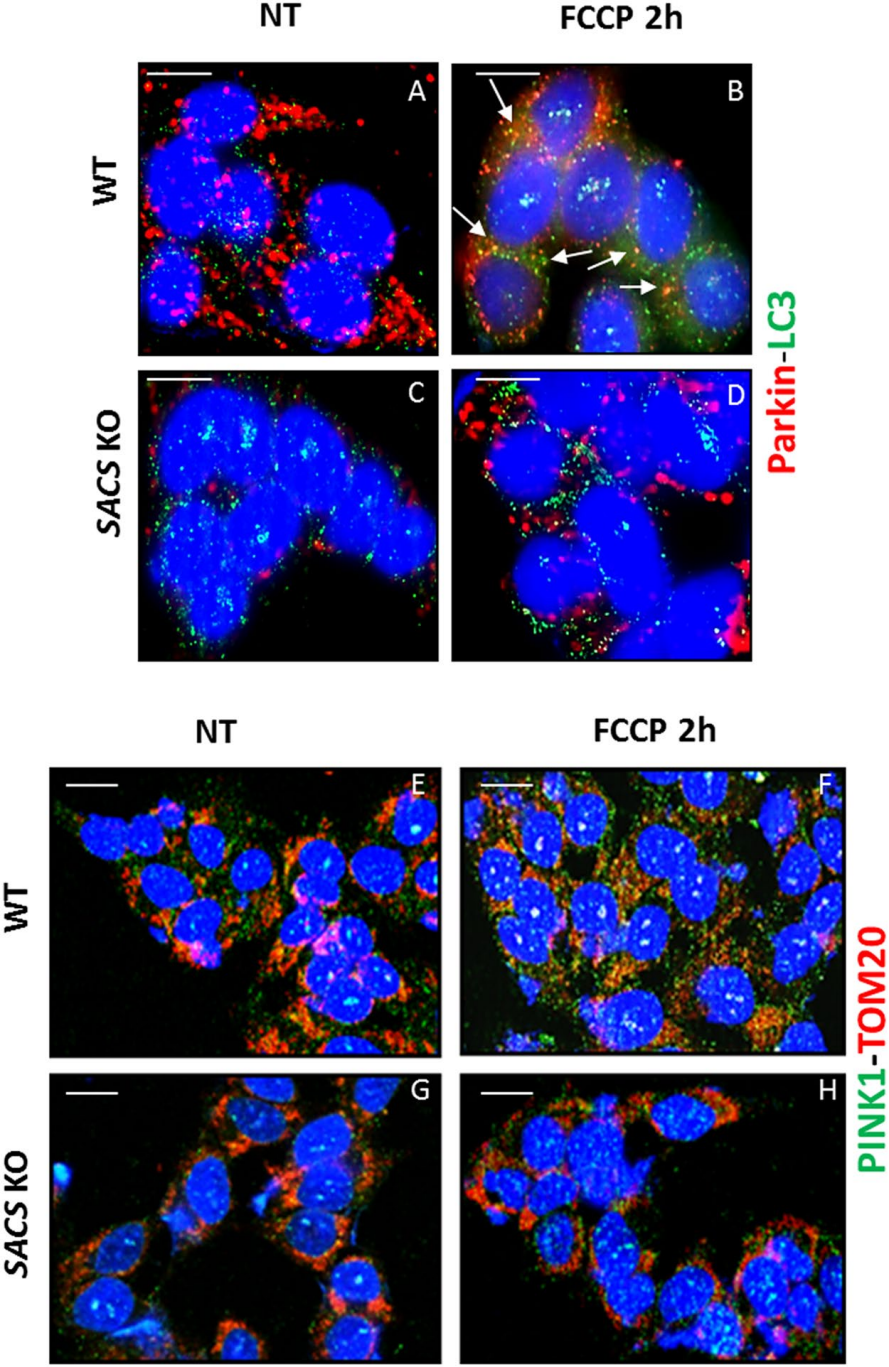
Mitophagic flux is altered in sacsin KO cells. (**A**–**D**) Immunofluorescence images of the mitophagy-related protein Parkin (red) and LC3 (green) in WT and KO cells under normal conditions (**A,C**) or upon FCCP treatment (**B,D**). There was no fusion between damaged mitochondria and autophagosomes in sacsin KO cells. Co-localization of Parkin with LC3 (yellow) is arrow indicated. (**E–H**) Co-staining of PINK1 (green) and TOM20 (in red) in WT and KO cells under normal conditions (**E,G**) or FCCP treatment (**F,H**) indicated a reduced localization of PINK1 on the outer mitochondrial membrane in KO cells after FCCP treatment. DAPI (in blue) was used as nuclear stain. Scale bar = 10 µm.

**Figure 9:**
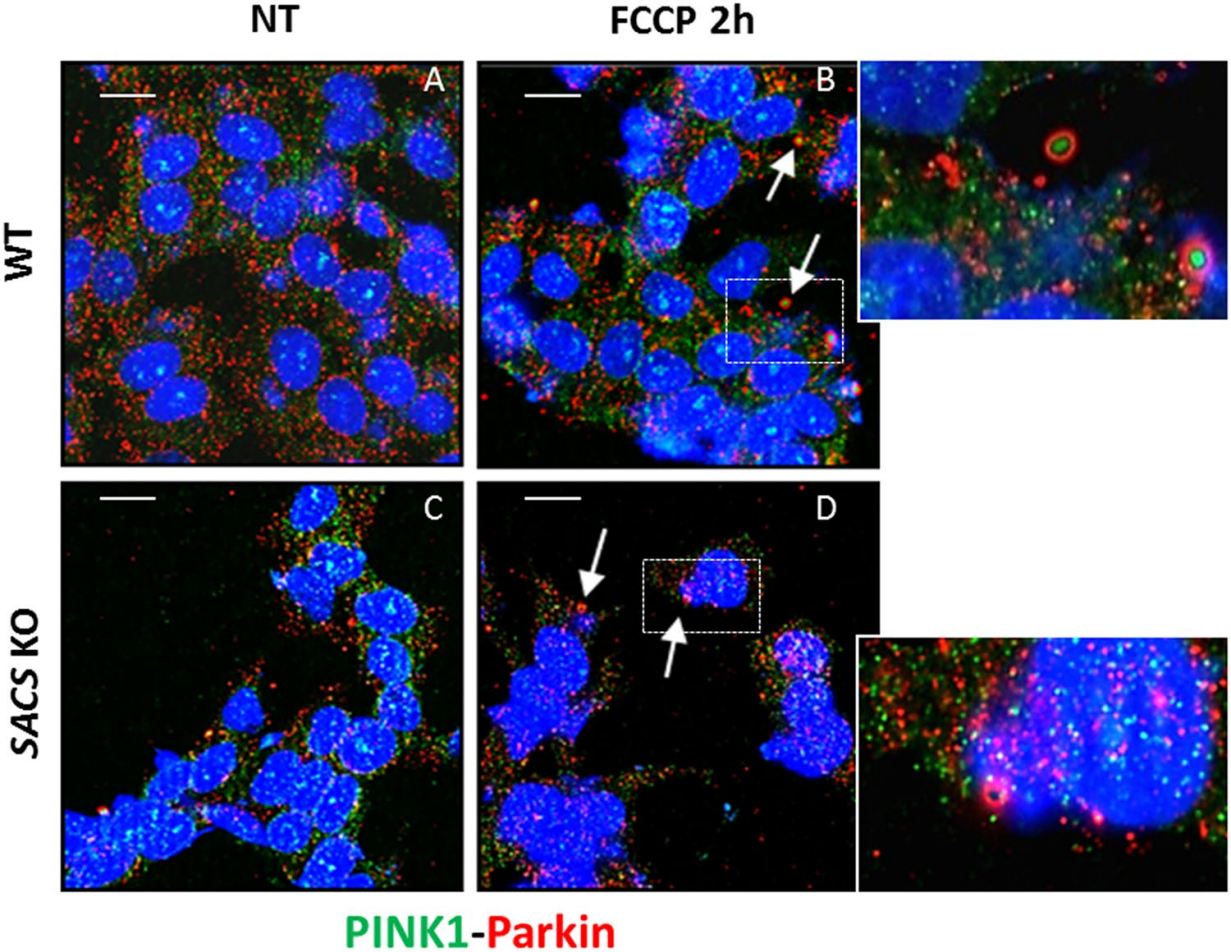
Mitophagic flux is damaged in sacsin KO cells. (**A,B**) Representative images of WT and KO cells immunolabeled for PINK1 (green) and Parkin (red) under normal conditions (**A,C**) or FCCP treatment (**B,D**) showed loss of co-localization of PINK1 with Parkin in KO cells compared to WT cells, indicating a defective mitophagic flux. Co-localization of PINK1 with Parkin (rounded structures) is arrow indicated (the insert shows an enlargement of these structures). DAPI (in blue) was used as nuclear stain. Scale bar = 10 µm.

To corroborate our hypothesis of mitophagic flux impairment, we also analyzed colocalization of LC3 with the mitophagy-related protein Parkin. During FCCP treatment, LC3 puncta colocalized with Parkin in WT but not in KO cells (Fig. 8B,D). This suggests that autophagosome recruitment of Parkin-labeled damaged mitochondria, prior to degradation of the latter in lysosomes, is impaired in the absence of sacsin. We also observed impaired recruitment of PINK1 after FCCP treatment in KO (Fig. 8H) as compared to WT cells (Fig. 8F). There was reduced co-localization of PINK1 with Parkin (Fig. 9D) and less pronounced rounded PINK1 aggregates with Parkin halo (compare Fig. 9B,D), all suggestive of a defective removal of damaged mitochondria.

### Prolonged autophagy induction by rapamycin restores correct autophagic flux in sacsin KO cells

**Figure 10:**
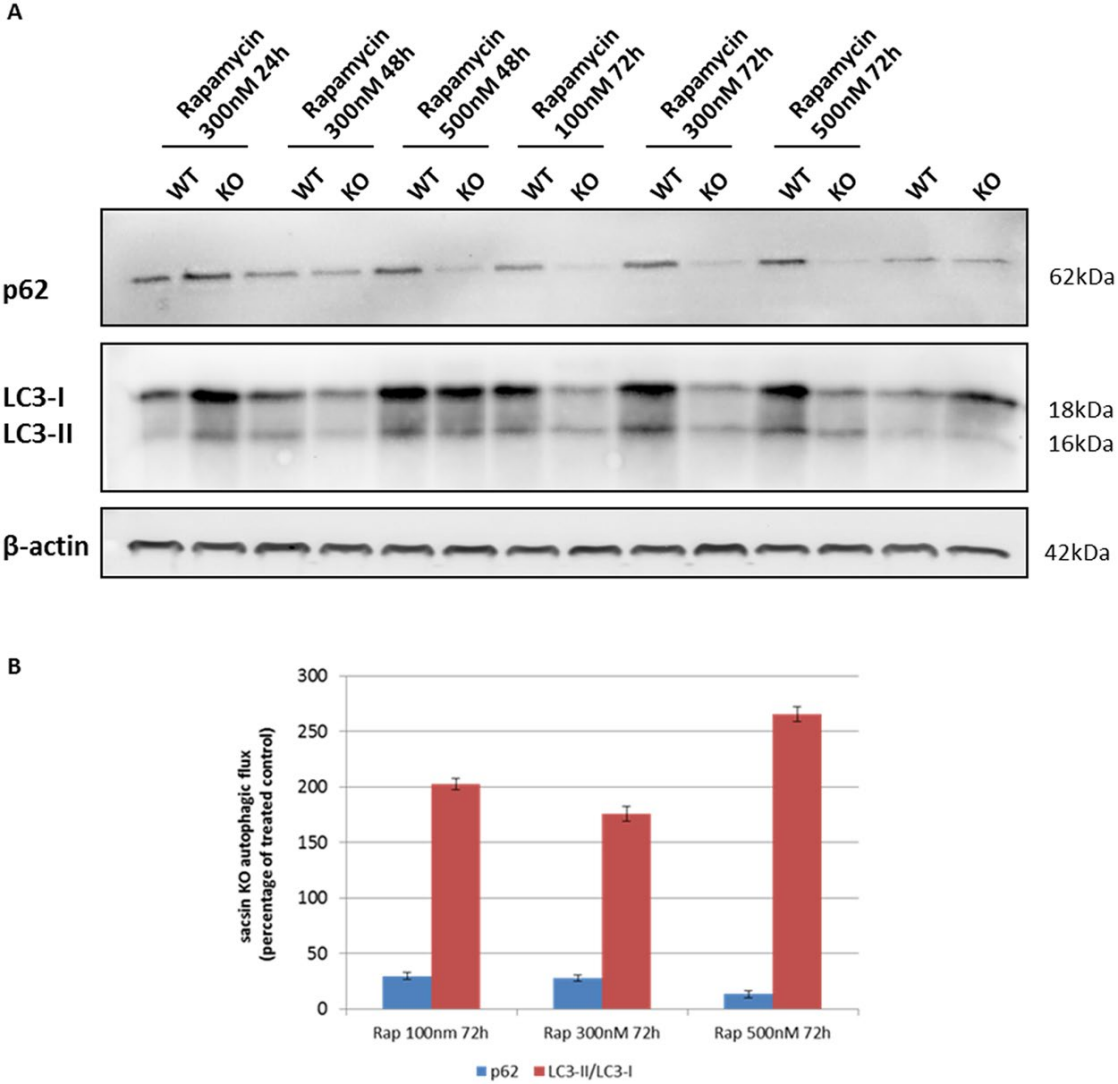
Rapamacyin-induced autophagy restores correct autophagic flux in KO cells after 72 h of treatment. (**A**) Western blotting analysis of the autophagy markers LC3 and p62 in WT and KO cells treated with different concentrations of the autophagy inducer rapamycin for up to 72 h. Full-length blots are presented in the Supplementary Information 5. (**B**) Trend of KO *vs* WT cells in rapamycin treatment at 72 h showed effective autophagic flux rescue. Data shown in this figure were reproduced independently three times. ns, not statistically significant; *p < 0.05; **p < 0.01.

Reactivation of autophagy by nutritional and pharmacological approaches in murine models of different neuromuscular disorders (e.g., *Col6a1*^−/−^ mice^30^) offers new possibilities in terms of autophagy-targeted therapeutic approaches. In our study, by measuring the LC3-II/LC3-I ratio^31^ we obtained proof-of-principle evidence that rapamycin, an FDA-approved inducer of autophagy already used in clinical trials and in neurodegenerative diseases^32^, can restore correct autophagic flux in a time- and dose-dependent manner in our cellular model of ARSACS (Fig. 10). Under rapamycin treatment, sacsin KO cells, more than WT ones, showed a higher LC3-II/LC3-I ratio with the highest values recorded with treatment at 500 nM for 72 h (Fig. 10A,B). Interestingly, p62 levels decreased upon 72-h treatment in KO cells (Fig. 10A,B), demonstrating effective rescue of the complete autophagic process.

## Discussion

Loss-of-function mutations in *SACS* lead to ARSACS and impair the function of sacsin, a protein that shares features with molecular chaperones^11,12,14^ and is implicated in mitochondrial dynamics^18–20^, neurofilament organization and protein quality control^22^. However, the mechanism underlying mutant sacsin-induced cell death is not fully understood. It is tempting to hypothesize that disrupted autophagy could be the element reconciling the chaperone-like function of sacsin with its role in mitochondrial dynamics. Other neurological diseases affecting both cerebellar and motor functions — cerebellar and motor impairments are major clinical features of ARSACS — are related to proteins that play a key role in early disruption of autophagy programs^13,33,34^ or are associated with key players in autophagic lysosome reformation at the intersection between endocytosis and autophagy^35,36^. Our working hypothesis was that the mediation of selective macroautophagy by molecular chaperones helps to explain the possible chaperone function of sacsin and its role in mitophagy. Indeed the selectivity of CASA is attributed to the chaperone Hsc70, which binds cargo proteins along with the small heat shock proteins HspB8 and Bag3^37^. Sacsin, through its aforementioned DnaJ domain^11^, interacts with ligases ubiquitinating cargos such as parkin linked to proteasomal protein degradation and to autophagy or mitophagy^38^.

In this study, RNA-seq revealed significant DEGs involved in oxidative phosphorylation and mitochondrial dynamics, and also in autophagy and cell death, thereby highlighting the important contribution made by sacsin to the cellular quality control system. After excluding alterations of the ubiquitin-proteasome system, the present study focused on the autophagy process. Our investigations in model cells genetically depleted of sacsin demonstrated that LC3-II expression levels were normally enhanced during FCCP treatment in control cells but limited in KO ones, whereas p62 levels were similarly decreased in both cell lines, suggesting that autophagy induction takes place in ARSACS. Cells without sacsin showed reduced autophagosome accumulation and autophagosome-lysosome fusion, and subsequent failure of cargo degradation; these cells contributed to mitophagy-induced fragmentation of mitochondria during FCCP treatment.

**Figure 11:**
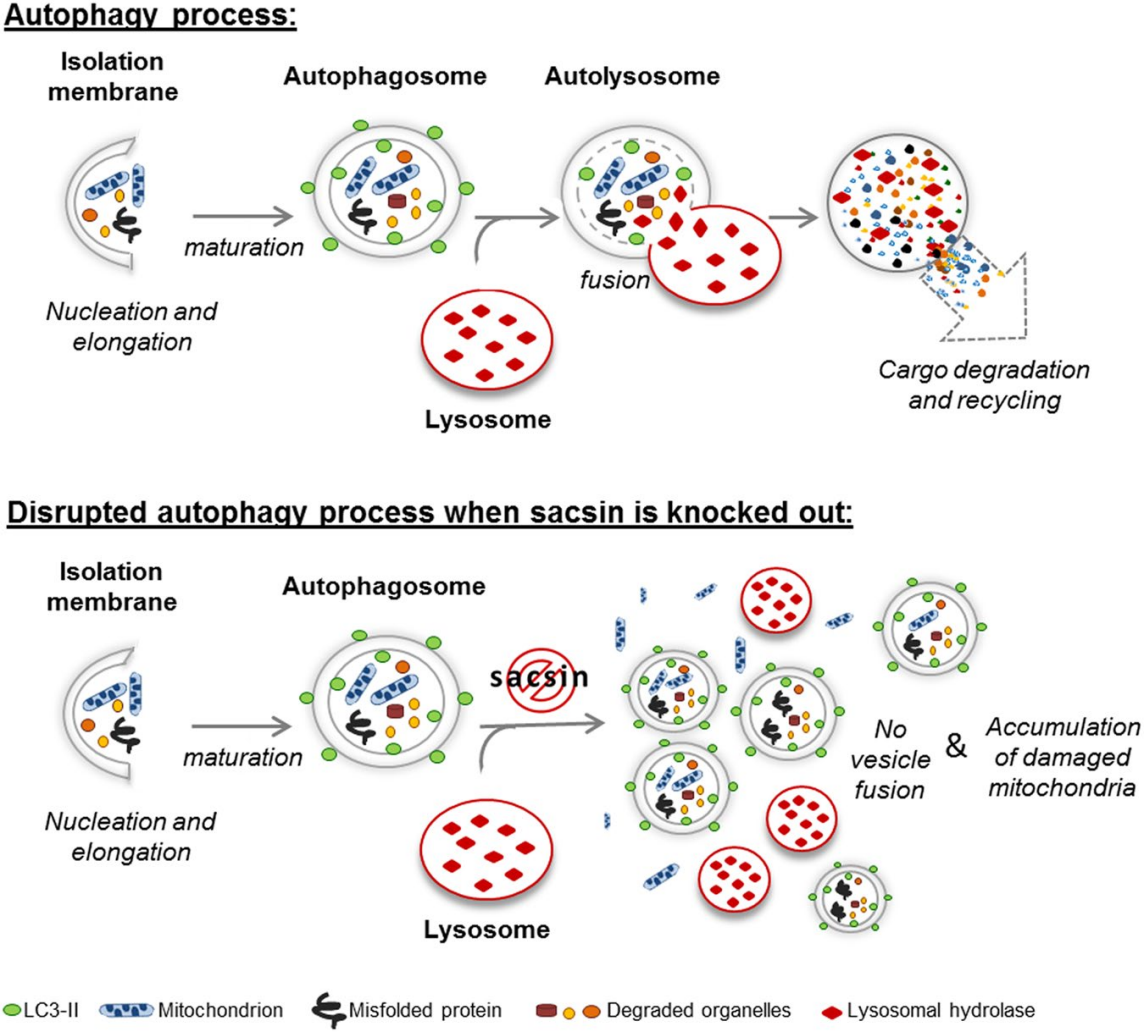
Scheme of autophagy processes in WT and sacsin KO cell models. Autophagy is active at a basal level serving for the turnover of long-lived proteins and also for the removal of superfluous or damaged organelles, such as mitochondria. The autophagy cascade consists of: formation of autophagosomes, their fusion with lysosomes to create autolysosomes and finally cytoplasmic cargo degradation by lysosomal hydrolases and recycling of macromolecules for the synthesis of essential components, to overcome various stress conditions. In our cell models, in which sacsin was knocked out, the fusion step did not take place. This resulted in accumulation of autophagosomes and damaged mitochondria in the cytoplasm.

In view of the above findings it seems plausible to expect autophagic flux defects when sacsin is not functioning. Two additional pieces of evidence further reinforce this hypothesis. First, knocking out sacsin in neuronal-like cell models results in increased oxidative stress and significant mitochondrial energy impairment and a block in the process of autophagosome-lysosome fusion. During FCCP treatment, failure of LC3-II to increase in the presence of bafilomycin A1 and p62 accumulation (Fig. 5) indicated a defective autophagy process in KO cells. By investigating co-localization of autophagy markers, we found that loss of sacsin disrupts autophagosome-lysosome fusion and selective mitochondria degradation, meaning that the autophagy process is impaired in its last steps (Fig. 11). Second, it was found that failure of the autophagy process in our ARSACS model can be modified pharmacologically. Indeed, we observed rapamycin-mediated autophagy reactivation, which appeared in a time- and dose-dependent manner. Besides validating an additional cell model of sacsin ablation, the results of the present study suggest that sacsin is a component of the network controlling autophagy and therefore that its loss disrupts the machinery of this process. Although data are preliminary, it is tempting to speculate that re-activation of autophagy opens up new opportunities for future approaches in ARSACS as already demonstrated in other inherited neurodegenerative diseases in both mice^39^ and men^40,41^.

In summary, our findings suggest that efficient autophagic flux in ARSACS is crucial for the clearance of damaged organelles and maintenance of homeostasis to promote neuronal survival. The impaired autophagic flux, inefficient degradation system, and subsequent impairment of mitochondrial accumulation observed in the neuronal-like KO cells appear to be linked to sacsin loss of function. The question of whether long-term pharmacological induction of autophagy (as performed using rapamycin in our study) could rescue motor and cerebellar functions *in vivo* remains an open issue to be considered in the cocktail for treating ARSACS.

## Materials and Methods

### Cell culture and treatments

Neuronal-like SH-SY5Y cells, a well-known cellular model for the experimental studies in neurodegenerative disease^42,43^, also used as model to study ARSACS^18^, were kindly donated by Prof. Ciro Isidoro, University of Piemonte Orientale, Novara, Italy. These cells were grown in Eagle’s Minimum Essential Medium (MEM) mixed in a 1:1 ratio with Ham’s F12 medium (Sigma-Aldrich). Cells were maintained in medium supplemented with 10% heat-inactivated FBS, 2mM L-glutamine, 100 U/ml penicillin, and 100 U/ml streptomycin (all from Euroclone S.p.A.). All cells were kept at 37 °C in a constant humidified 5% CO_2_ atmosphere. For the experiments, growing cells were plated on sterile plastic dishes or on sterile glass coverslips and allowed to adhere for at least 24 h before use. For inhibition of autophagy, bafilomycin A1 was added to cells at a concentration of 200 nM 2 h before the end of the treatment. To induce autophagy rapamycin was added to cells at concentrations of 100 nM, 300 nM and 500 nM for up to 72 h. To inhibit autophagy 3-MA was added at a concentration of 10 mM for at least 3 h. The uncoupler FCCP was used at 20 µM for 2 h. All chemicals came from Sigma-Aldrich.

### Generation of sacsin KO cell lines using the CRISPR/Cas9 system

The sacsin CRISPR guide RNA (gRNA) sequences were designed in order to efficiently target the *SACS* gene (NM_014363) with minimal risk of off-target Cas9 binding elsewhere in the genome, as suggested by others^44^. To this end, three gRNA constructs were selected: CTGGACCGTGCGCGATGTGA, exon 2; GACTCTTTGGTCAAAAGATA, exon 5; GAGGATCTAGCATCCCGATT, exon 7. The carrying plasmid expression vector was constructed by using the pSpCas9(BB)-2A-Puro (PX459) V2.0 plasmid (Addgene, Cambridge, MA) backbone, developed for CRISPR/Cas9 purposes and subsequent insertion of the single-guide RNA (sgRNA) sequences of interest. After insertion of the specific gRNA inside the plasmid, it was transformed into a competent *E. coli* strain (One Shot Stbl3 Chemically Competent *E. coli*; Invitrogen-ThermoFisher Scientific, Waltham, MA). A small number of colonies were subsequently collected to isolate the plasmid DNA (QIAprep® spin miniprep Kit; Qiagen, Hilden, Germany) checking for the correct insertion of a guide construct (annealed top and bottom strand of sgRNA) through U6 plasmid fragment amplification and sequencing. SH-SY5Y cell lines were then transfected with lipofectamine 3000 (Invitrogen-ThermoFisher Scientific) following the standard protocol. One-day post transfection, cells were grown in regular media supplemented with puromycin (5 µg/mL, from Sigma-Aldrich) for 48–72 h in order to select those that had incorporated the exogenous DNA. TIDE free software was used to quantify the editing efficiency and to identify the predominant types of insertions or deletions in the DNA of targeted cells. Once evaluated for overall editing efficiency, the transfection pools were used to isolate single clones by means of the “limiting-dilution technique”: the cell suspension was diluted and plated at a density of 0.3 cells/well (in a 96-well plate).

Genotypes and phenotypes of clones grown in wells under regular conditions (growing time about 3 weeks) were verified by standard sequencing methods and Western blotting analysis. The edited clones with lack of protein expression were expanded.

### RNA-sequencing data-production and analysis

Whole transcriptome analysis was performed by RNA-seq technology on WT and sacsin KO SH-SY5Y cells as previously reported^45^. Briefly, cells were collected in three independent experiments and total RNA was isolated using TripleXtractor (Grisp Research Solutions, Porto, Portugal). RNA purity and quantity were checked using Nanophotometer^TM^ Pearl-version 1.2 (IMPLEN, Westlake Village, CA), while RNA integrity (RNA integrity number ≥8.0) was assessed using the RNA 6000 Nano Kit (Agilent Technologies, Santa Clara, CA). Directional RNA-seq libraries were prepared from 1500 ng total RNA using the TruSeq Stranded kit (Illumina, San Diego, CA) after poly(A) capture, according to the manufacturer’s instructions. Libraries were quantified by real-time PCR, pooled at equimolar concentration, and sequenced on an Illumina NextSeq500 sequencer (Illumina), applying standard manufacturer protocols. About 30 million single-end (75 bp) reads were generated for each sample. The quality of reads obtained from each sample was assessed using FastQC software, and reads with more than 10% of undetermined bases or more than 50 bases with a quality score <7 were discarded, as reported^45^. Reads were then clipped from adapter sequences using Scythe software version 0.980, and low-quality ends (Q score <20 on a 10-nt window) were trimmed with Sickle version 0.940. Alignment of reads to a reference human genome (GRCh38) was performed using HISAT2 version 2.0.1.

Differential gene expression analysis between WT and KO cells was performed with DESeq2 version 1.16.1. The normalized expression values expressed as fragments per kilobase per million mapped reads for each gene were calculated according to the formula ((count/(total reads/10^6^))/(gene length/10^3^)). Genes showing an adjusted p-value ≤ 0.05 were assigned as differentially expressed.

### Pathway enrichment analysis

Differentially expressed genes (corresponding to identified transcripts) were evaluated by online TOPPFUN enrichment analysis or iPathwayGuide analysis (Advaita Bioinformatics) to recognize significantly enriched biological processes and molecular pathways. Only the most meaningful functional annotations, showing the lowest p values, were evaluated; in addition, only scores ≥+1 (predicted activation) or ≤−1 (predicted inhibition) were considered as in ref.^45^.

### Aggresome detection

Cellular aggresomes were detected using the ProteoStat® Aggresome Detection kit (Enzo LifeSciences, Lausanne, Switzerland), containing a specific 488 excitable red fluorescent molecular rotor dye, following previously described protocols^46^. Cultured cells were grown on coverslips and incubated for 12 h with MG-132, a cell-permeable proteasome inhibitor (5 μM), which was used as a positive control. The cells were then washed with Phosphate buffered saline (PBS), fixed in 4% paraformaldehyde for 30 min at room temperature, and permeabilized in experimental solution (0.5% Triton X-100, 3 mM Ethylenediaminetetraacetic acid (EDTA), pH 8.0 in 1X assay buffer) with gentle shaking on ice for 30 min. The cells were washed again with PBS and incubated in a blocking solution (FBS 20% in PBS1X) for 1 h at room temperature. Overnight incubation in a cold room was performed with the mouse monoclonal anti-p62 primary antibody (BD Transduction Laboratories^TM^, San Jose, CA; dilution 1:200). The next day cells on coverslips were washed with PBS and incubated for 1 h at room temperature in a humid chamber with the secondary antibody: goat anti-mouse conjugated with AlexaFluor 488 (Cell Signaling Technology Inc., Danvers, MA). After this step, the cells were washed again with PBS and stained using the ProteoStat® Aggresome Detection Reagent and Hoechst 33342 nuclear stain (Invitrogen-ThermoFisher Scientific) for 30 min at room temperature protected from the light. The aggresomes were visualized using a Zeiss AX10 inverted fluorescence microscope equipped with an AxioCam MRc5 camera (Zeiss, Munich, Germany). The images were processed using AxioVision rel 4.8 acquisition software (Zeiss).

### Immunofluorescence stains

The cells adherent on sterile glass coverslips, previously treated with Poly-D-Lysine (Sigma-Aldrich) were fixed in cold methanol for 20 min and permeabilized with 0.1% Triton X-100 in PBS1X for 15 min. Coverslips were washed in PBS1X and incubated in a blocking solution (FBS 20% in PBS1X) for 1 h at room temperature. Overnight incubation in a cold room was performed with the following primary antibodies: mouse monoclonal anti-p62 (BD Transduction Laboratories^TM^, San Jose, CA; dilution 1:200); rabbit polyclonal anti-TOM20 (Santa Cruz Biotechnology Inc., Santa Cruz, CA; dilution 1:100); mouse monoclonal anti-Human LAMP1 (BD Pharmingen^TM^, San Jose, CA; dilution 1:1000); mouse monoclonal anti-Parkin (Abcam, Cambridge, England; diluition 1:100); mouse monoclonal anti-Vimentin (Abcam, Cambridge, England; diluition 1:100); rabbit polyclonal anti-LC3 (Sigma-Aldrich; dilution 1:1000); and rabbit polyclonal anti-PINK1 (Abcam, Cambridge, England; diluition1:1000). As secondary antibodies (dilution 1:1000), goat anti-mouse or anti-rabbit antibodies conjugated with AlexaFluor 488 or AlexaFluor 555 dye (Cell Signaling Technology Inc., Danvers, MA) were used as appropriate for 1 h at room temperature in a humid chamber. Nuclear chromatin was stained with the fluorescent dye 4,6-diamidino- 2-phenylindole-dihydrochloride 5 µg/ml (DAPI, Sigma-Aldrich). Stained cells were mounted for microscopy. Images were acquired by a Zeiss AXIO IMAGER.M2 fluorescence microscope equipped with an AxioCam MRc5 camera using a 63X/1.4 oil objective and processed by AxioVision release 4.8.2 acquisition software (Zeiss).

For staining of mitochondria with MitoTracker Red (Invitrogen-ThermoFisher Scientific), the stock solution was diluted to a concentration of 200 nM in cell culture media before being added to cells and left for 30 min at 37 °C in 5% CO_2_ atmosphere. After the incubation period, cells were washed twice in PBS prior to live imaging or fixation as in reference^20^.

### Western blotting

For Western blotting, SH-SY5Y stable clones were collected at confluence, washed twice with PBS and then homogenized in RIPA buffer (150 mM NaCl, 50 mM Tris–HCl, 6 mM EDTA, 1% NP-40, 0.1% SDS, 0.5% deoxycolic acid, pH 8.0) containing inhibitors of proteases (Roche Diagnostics GmbH, Mannheim, Germany), following standard protocols. The cells were disrupted by plastic pestle on ice and centrifuged for 10 min at 16,000 × g at 4 °C.

Fifteen to fifty μg of cell proteins, determined by BCA assay (Invitrogen-ThermoFisher Scientific), were denatured, separated by electrophoresis using precast NuPAGE™ 3–8% Tris-Acetate gel or Novex 8–16% Tris-Glycine Mini Gels (Invitrogen-ThermoFisher Scientific) and then electro-blotted onto PVDF membranes (Bio-Rad Laboratories Inc., Hercules, CA). Membranes were blocked with TBS/0.1%-Tween20 (TTBS) containing 5% non-fat dry milk before overnight incubation with the specified antibodies. Primary antibodies were incubated overnight at 4 °C in TTBS with 2.5% non-fat dry milk, and those not bound specifically were removed by washing in TTBS. The following primary antibodies were used: rabbit polyclonal anti-sacsin (EMD Millipore, Temecula, CA); mouse monoclonal anti-SDH70 (MitoSciences Inc., Eugene, OR); rabbit polyclonal anti-phospho-S6 (Cell Signaling Technology Inc.); rabbit polyclonal anti-S6 (Cell Signaling Technology Inc.); mouse monoclonal anti-p62 (BD Transduction Laboratories^TM^, San Jose, CA); rabbit polyclonal anti-LC3 (Sigma-Aldrich); and mouse monoclonal anti-MFN2 (Abnova, Taiwan). The filter was subsequently stripped and re-probed with an antibody specific for GAPDH (Abcam) or β–actin (Cell Signaling Technology Inc.) as an index of homogenate protein loading in the lanes. Peroxidase-conjugated anti-mouse and anti-rabbit secondary antibodies (Jackson ImmunoResearch, laboratories Inc., West Grove, PA) were added for 1 h at room temperature in the same buffer as used for the primary antibodies (2.5% non-fat dry milk in TTBS). Reactive bands were detected using Clarity Max^TM^ Western ECL Substrate (Bio-Rad Laboratories Inc.), according to the manufacturer’s instructions. Densitometry of Western blot bands was performed with the ImageLab 6.0 software (Bio-Rad Laboratories Inc.) and with the open source Image J software.

### Oxygen consumption rate (OCR) measurement

Measurements of OCR were performed using a Seahorse XF^e^24 Extracellular Flux Analyzer (Agilent Technologies) as in^15^. Cells were counted in an automated cell counter (TC20, Bio-Rad Laboratories Inc.), seeded in XF24 microplates (Agilent Technologies) at a density of 60000 cells/well for SH-SY5Y, and incubated overnight at 37 °C in a 5% CO_2_ atmosphere. After an incubation at 37 °C in a non-CO_2_ incubator for 1 h, OCR was measured in XF media (non-buffered DMEM medium, pH 7.4, containing 10 mM glucose, 2 mM L-glutamine and 1 mM sodium pyruvate), under basal conditions and in response to 2 µM oligomycin, 1.5 µM FCCP and 1 µM antimycin A and rotenone (all chemicals from Sigma-Aldrich). Data were normalized for cell number.

### Detection of cellular reactive oxygen species

Fluorimetric determination of intracellular ROS was performed using a DCFDA assay kit (Abcam). Harvested cells in a 96-well microplate were incubated with 25 µM DCFDA in 1X Buffer for 45 min in the dark at 37 °C. Upon removal of DCFDA solution, 100 μL/well of 1X Buffer or 1X PBS was added and the fluorescence was measured immediately. For toxicity assays, 100 μL/well of TBHP 150 µM solution was added and cells were incubated for 1 h in the dark at 37 °C. For each sample, 10,000 events were acquired and intracellular ROS formation, which results from oxidation of the reagent, was detected by fluorescence spectroscopy, with maximum excitation and emission spectra of 495 nm and 529 nm respectively. Cells were analyzed using a GloMax® multimode plate reader (Promega Corporation, Madison, WI). The data were analyzed in triplicate.

### Mitochondrial membrane potential measurements

Neuroblastoma cell lines were plated at a density of 50000 cells/well in a 96-well plate and grown in regular medium for 24 hours. Mitochondrial membrane potential (Δψm) was assessed with TMRM (Invitrogen, Carlsbad, CA) to label active mitochondria. TMRM is a cell permeant, positively-charged fluorescent dye, which accumulates in active mitochondria in a potential-dependent manner. Depolarized or inactive mitochondria fail to take up the probe which remains scattered in the cytosol. The dye was loaded onto cells at 100 nM in Hank’s Balanced Salt Solution supplemented with 10 mM HEPES, 2 µM cyclosporine H, pH 7.4, at 37 °C for 5 minutes; the cells were then washed three times in PBS and fluorescence intensity was measured using a Spectramax iD3 microplate reader (Molecular Devices, San Jose, CA; excitation: 544 nm, emission: 590 nm, bottom reading). To verify the specificity of TMRM-related signal, a parallel assay was carried out using a depolarization control (20 μM FCCP as uncoupling agent). All data were expressed as specific TMRM fluorescence intensity and normalized by DAPI staining as a function of cell number (all chemicals from Sigma-Aldrich).

### Statistics

If not otherwise specified, all experiments were replicated independently three times. Data are given as average values ± SD. Statistical significance was calculated using Student’s t parametric test set at: *p < 0.05; **p < 0.01; and ***p < 0.001.

## Supplementary information

Supplementary Information file

## Data Availability

All data generated or analyzed during this study are included in this published article and its Supplementary Information files. Sequencing data have been deposited in NCBI under Bioproject accession number PRJNA548751. RNA-seq raw data are accessible through Sequence Read Archive (SRA) accession number SRR9302756-61.

## References

[1] Yamamoto Y (2005). Novel compound heterozygous mutations in sacsin-related ataxia. J. Neurol. Sci..

[2] Takiyama Y (2006). Autosomal recessive spastic ataxia of Charlevoix-Saguenay. Neuropathology.

[3] Vermeer S (2008). ARSACS in the Dutch population: a frequent cause of early-onset cerebellar ataxia. Neurogenetics.

[4] Synofzik M (2013). Autosomal recessive spastic ataxia of Charlevoix Saguenay (ARSACS): expanding the genetic, clinical and imaging spectrum. Orphanet J. Rare Dis..

[5] Baets J (2010). Mutations in SACS cause atypical and late-onset forms of ARSACS. Neurology.

[6] Bouchard JP, Barbeau A, Bouchard R BR (1978). Autosomal Recessive Spastic Ataxia of. Can J Neurol Sci.

[7] Engert JC (2000). ARSACS, a spastic ataxia common in northeastern Québec, is caused by mutations in a new gene encoding an 11. 5-kb ORF. Nat. Genet..

[8] Yu-Wai-Man P (2014). Abnormal retinal thickening is a common feature among patients with ARSACS-related phenotypes. Br. J. Ophthalmol..

[9] Bouhlal Y, Amouri R, El Euch-Fayeche G, Hentati F (2011). Autosomal recessive spastic ataxia of Charlevoix-Saguenay: an overview. Parkinsonism Relat. Disord..

[10] Thiffault I (2013). Diversity of ARSACS mutations in French-Canadians. Can. J. Neurol. Sci..

[11] Parfitt DA (2009). The ataxia protein sacsin is a functional co-chaperone that protects against polyglutamine-expanded ataxin-1. Hum. Mol. Genet..

[12] Anderson JF, Siller E, Barral JM (2010). The Sacsin Repeating Region (SRR): A Novel Hsp90-Related Supra-Domain Associated with Neurodegeneration. J. Mol. Biol..

[13] Greer PL (2010). The Angelman Syndrome protein Ube3A regulates synapse development by ubiquitinating arc. Cell.

[14] Anderson JF, Siller E, Barral JM (2011). The neurodegenerative-disease-related protein sacsin is a molecular chaperone. J. Mol. Biol..

[15] Kozlov G (2011). Structural basis of defects in the sacsin HEPN domain responsible for Autosomal Recessive Spastic Ataxia of Charlevoix-Saguenay (ARSACS). J. Biol. Chem..

[16] Li X (2015). High-throughput screening for ligands of the HEPN domain of sacsin. PLoS One.

[17] Ménade Marie, Kozlov Guennadi, Trempe Jean-François, Pande Harshit, Shenker Solomon, Wickremasinghe Sihara, Li Xinlu, Hojjat Hamed, Dicaire Marie-Josée, Brais Bernard, McPherson Peter S., Wong Michael J. H., Young Jason C., Gehring Kalle (2018). Structures of ubiquitin-like (Ubl) and Hsp90-like domains of sacsin provide insight into pathological mutations. Journal of Biological Chemistry.

[18] Girard M (2012). Mitochondrial dysfunction and Purkinje cell loss in autosomal recessive spastic ataxia of Charlevoix-Saguenay (ARSACS). Proc. Natl. Acad. Sci..

[19] Larivière R (2015). Sacs knockout mice present pathophysiological defects underlying autosomal recessive spastic ataxia of charlevoix-saguenay. Hum. Mol. Genet..

[20] Bradshaw TY (2015). A reduction in Drp1-mediated fission compromises mitochondrial health in autosomal recessive spastic ataxia of Charlevoix Saguenay. Hum. Mol. Genet..

[21] Criscuolo C (2015). Powerhouse failure and oxidative damage in autosomal recessive spastic ataxia of Charlevoix-Saguenay. J. Neurol..

[22] Duncan EJ (2017). Altered organization of the intermediate filament cytoskeleton and relocalization of proteostasis modulators in cells lacking the ataxia protein sacsin. Hum. Mol. Genet..

[23] Kaushik S, Cuervo AM (2012). Chaperones in autophagy. Pharmacol. Res..

[24] Sahu R (2011). Microautophagy of cytosolic proteins by late endosomes. Dev. Cell.

[25] Wang Z, Gerstein M, Snyder M (2009). RNA-Seq: a revolutionary tool for transcriptomics. Nat. Rev. Genet..

[26] Wu Y-T (2010). Dual role of 3-methyladenine in modulation of autophagy via different temporal patterns of inhibition on class I and III phosphoinositide 3-kinase. J. Biol. Chem..

[27] Klionsky DJ (2016). Guidelines for the use and interpretation of assays for monitoring autophagy (3rd edition. Autophagy.

[28] Mizushima N, Yoshimori T, Levine B (2010). Methods in Mammalian Autophagy Research. Cell.

[29] Chen H, Chan DC (2009). Mitochondrial dynamics-fusion, fission, movement, and mitophagy-in neurodegenerative diseases. Hum. Mol. Genet..

[30] Grumati P (2010). Autophagy is defective in collagen VI muscular dystrophies, and its reactivation rescues myofiber degeneration. Nat. Med..

[31] Karim Md. Razaul, Kanazawa Takumi, Daigaku Yasuhiro, Fujimura Shinobu, Miotto Giovanni, Kadowaki Motoni (2007). Cytosolic LC3 Ratio as a Sensitive Index of Macroautophagy in Isolated Rat Hepatocytes and H4-II-E Cells. Autophagy.

[32] Rubinsztein DC, Gestwicki JE, Murphy LO, Klionsky DJ (2007). Potential therapeutic applications of autophagy. Nat. Rev. Drug Discov..

[33] Vantaggiato C (2013). Defective autophagy in spastizin mutated patients with hereditary spastic paraparesis type 15. Brain.

[34] Kim, M. *et al*. Mutation in ATG5 reduces autophagy and leads to ataxia with developmental delay. *Elife* **5** (2016). 10.7554/eLife.12245 PMC4786408 26812546

[35] Chang J, Lee S, Blackstone C (2014). Spastic paraplegia proteins spastizin and spatacsin mediate autophagic lysosome reformation. J. Clin. Invest..

[36] Varga R-E (2015). *In Vivo* Evidence for Lysosome Depletion and Impaired Autophagic Clearance in Hereditary Spastic Paraplegia Type SPG11. PLoS Genet..

[37] Arndt V (2010). Chaperone-Assisted Selective Autophagy Is Essential for Muscle Maintenance. Curr. Biol..

[38] Sarraf SA (2013). Landscape of the PARKIN-dependent ubiquitylome in response to mitochondrial depolarization. Nature.

[39] Johnson SC (2013). mTOR inhibition alleviates mitochondrial disease in a mouse model of Leigh syndrome. Science.

[40] Bissler JJ (2017). Everolimus long-term use in patients with tuberous sclerosis complex: Four-year update of the EXIST-2 study. PLoS One.

[41] Palavra F, Robalo C, Reis F (2017). Recent Advances and Challenges of mTOR Inhibitors Use in the Treatment of Patients with Tuberous Sclerosis Complex. Oxid. Med. Cell. Longev..

[42] Shastry P, Basu A, Rajadhyaksha MS (2001). Neuroblastoma cell lines–a versatile *in vitro* model in neurobiology. Int. J. Neurosci..

[43] Xicoy H, Wieringa B, Martens GJM (2017). The SH-SY5Y cell line in Parkinson’s disease research: a systematic review. Mol. Neurodegener..

[44] Sanjana NE, Shalem O, Zhang F (2014). Improved vectors and genome-wide libraries for CRISPR screening. Nat. Methods.

[45] Pezzini F (2017). Transcriptomic Profiling Discloses Molecular and Cellular Events Related to Neuronal Differentiation in SH-SY5Y Neuroblastoma Cells. Cell. Mol. Neurobiol..

[46] Jerič B (2013). N-terminally truncated forms of human cathepsin F accumulate in aggresome-like inclusions. Biochim. Biophys. Acta.

