# Supplementary material for: Functional Transcriptome Analysis in ARSACS KO Cell Model Reveals a Role of Sacsin in Autophagy
Source: Sci Rep. 2019 Aug 15;9:11878. doi: 10.1038/s41598-019-48047-x (PMC6695435; doi:10.1038/s41598-019-48047-x)
Supplement: Supplementary file 1 — Supplementary Information file [file 41598_2019_48047_MOESM1_ESM.pdf]

FUNCTIONAL TRANSCRIPTOME ANALYSIS IN ARSACS KO CELL MODEL REVEALS A ROLE OF SACSIN IN AUTOPHAGY

Federica Morani[1*], Stefano Doccini[1], Roberto Sirica[2], Marta Paterno[2], Francesco Pezzini[3], Ivana Ricca[1], Alessandro Simonati[3], Massimo Delledonne[2], Filippo Maria Santorelli[1*]

[1]Molecular Medicine for Neurodegenerative and Neuromuscular Diseases Unit, IRCCS Stella Maris Foundation, Pisa 56128, Italy;
[2]Department of Biotechnology, University of Verona, Verona 37134, Italy;
[3]Neurology (Neuropathology and Child Neurology), Department of Neuroscience, Biomedicine and Movement, University of Verona, Verona 37134, Italy

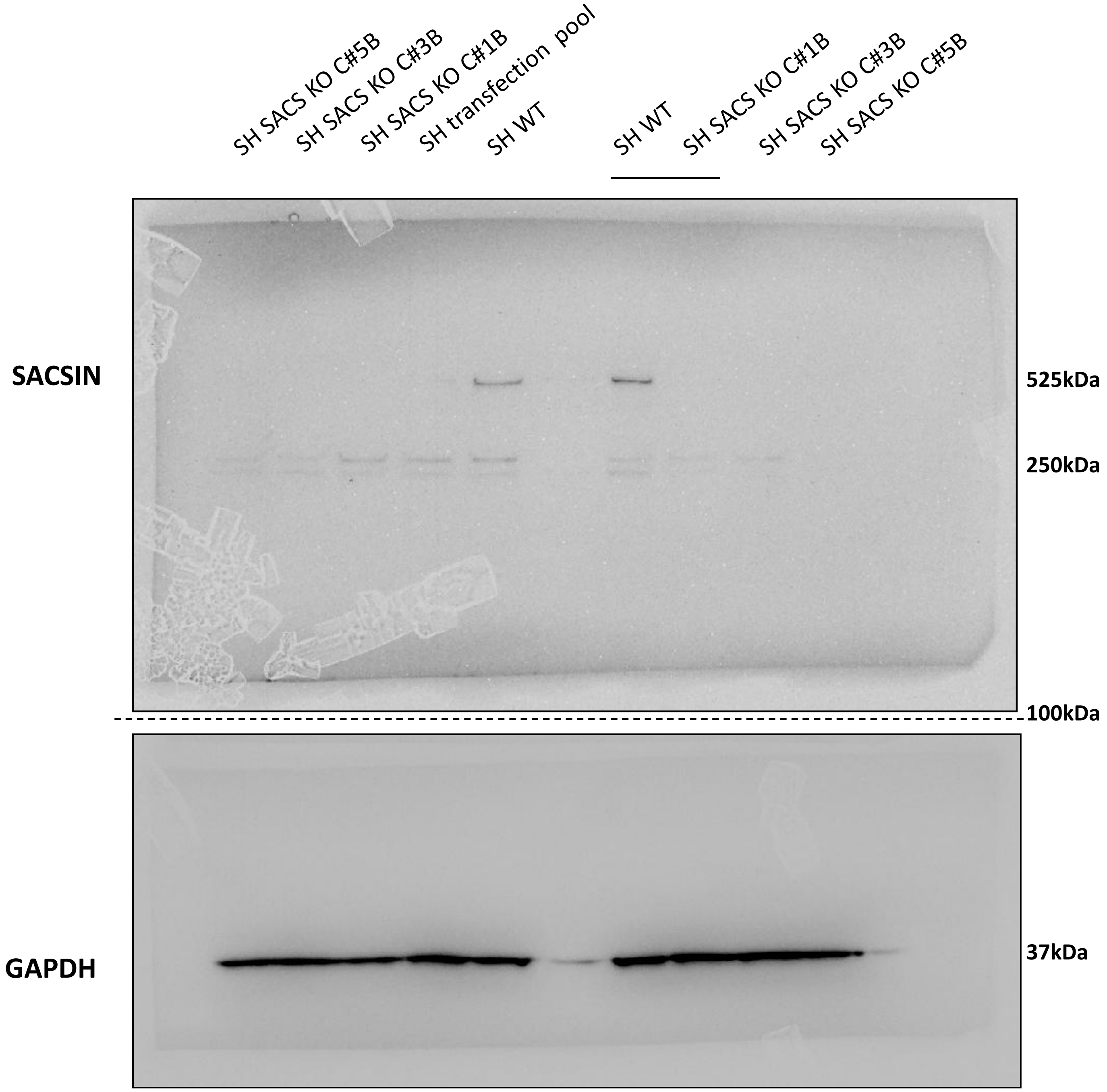

Supplementary Information 1. Full-length blots refer to Figure 1F and Supplementary Figure S1B. Dotted line indicates the cut on the PVDF-membrane.

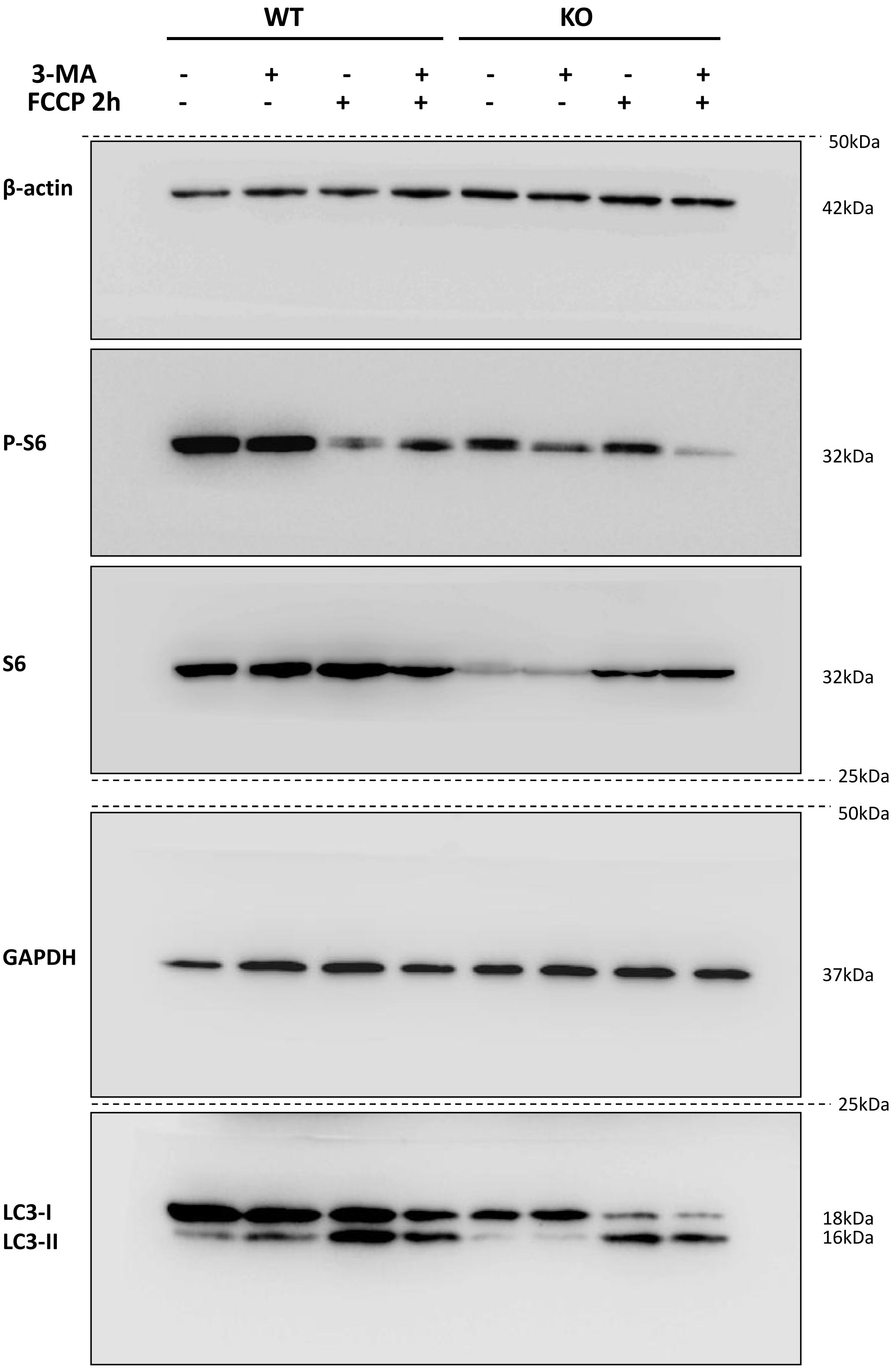

Supplementary Information 2. Full-length blots refer to Figure 4A. Dotted lines indicate the cut on the PVDF-membranes.

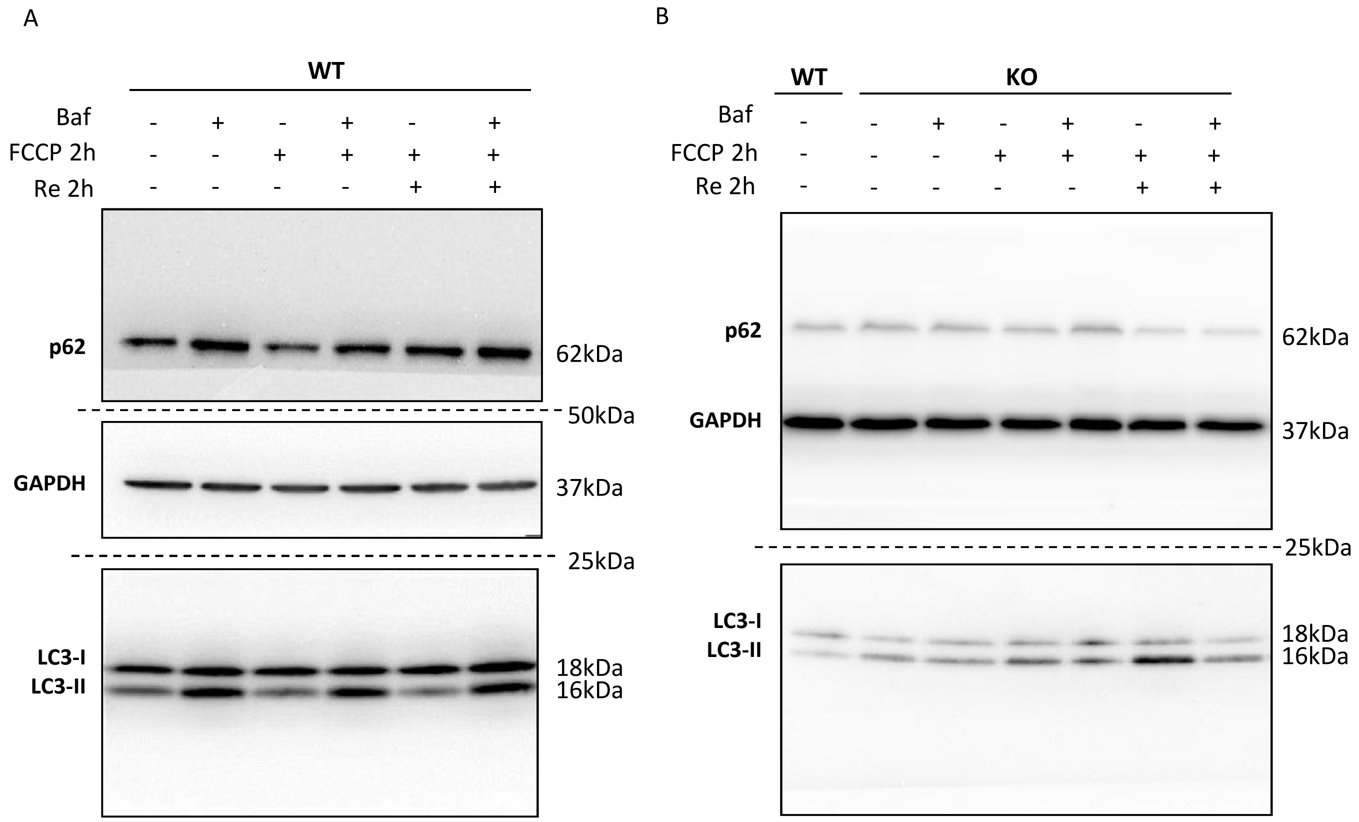

Supplementary Information 3. Full-length blots refer to Figure 5.
Dotted lines indicate the cut on the PVDF-membrane.

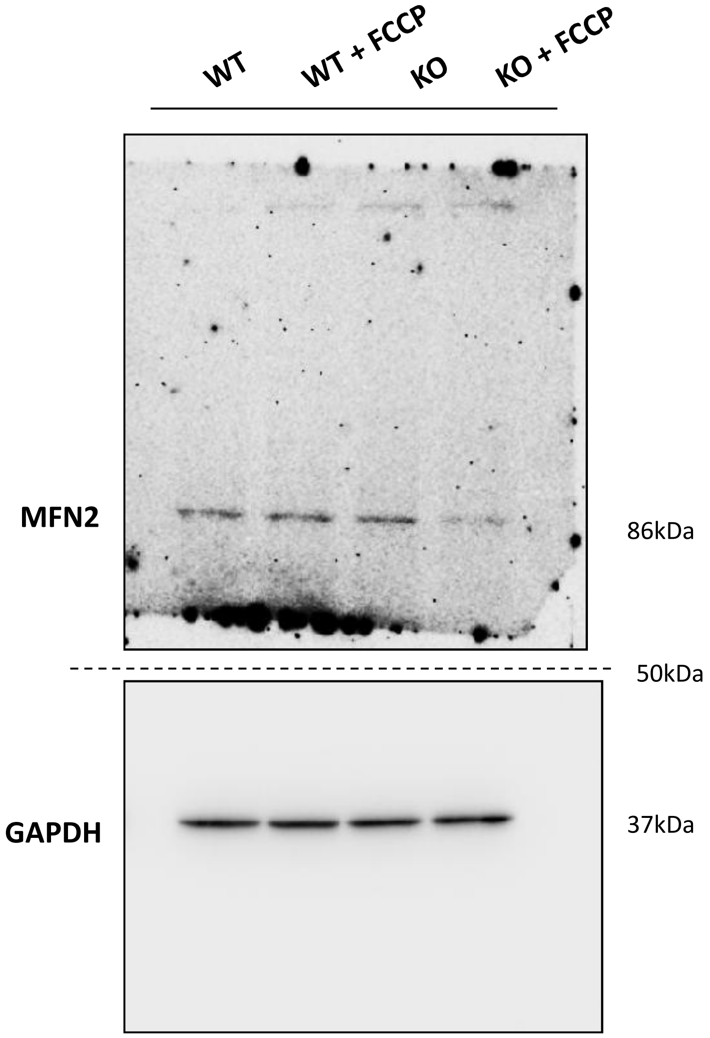

Supplementary Information 4. Full-length blots refer to Figure 7C. Dotted line indicates the cut on the PVDF-membrane.

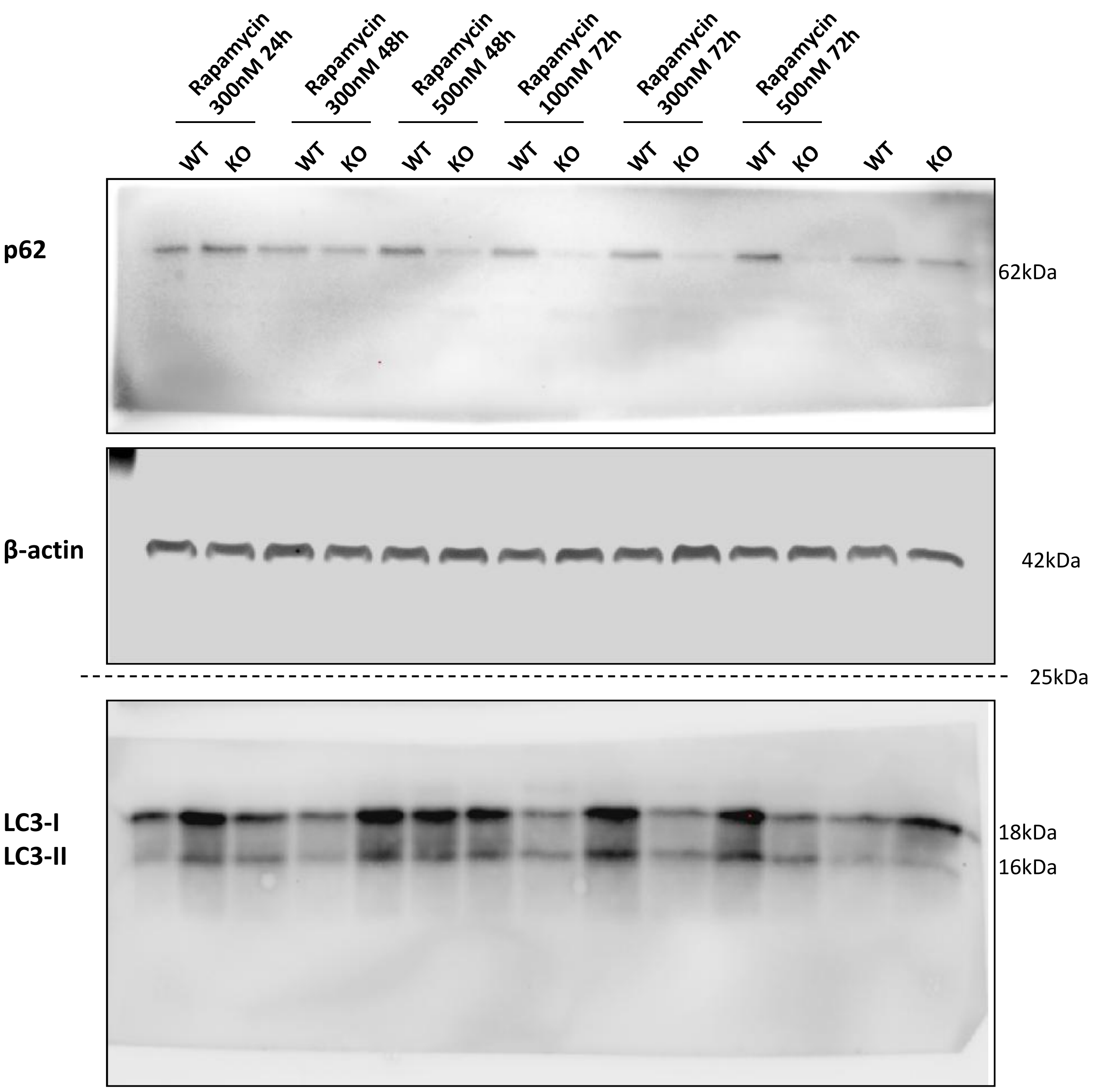

Supplementary Information 5. Full-length blots refer to Figure 10A. Dotted lines indicate the cut on the PVDF-membrane.

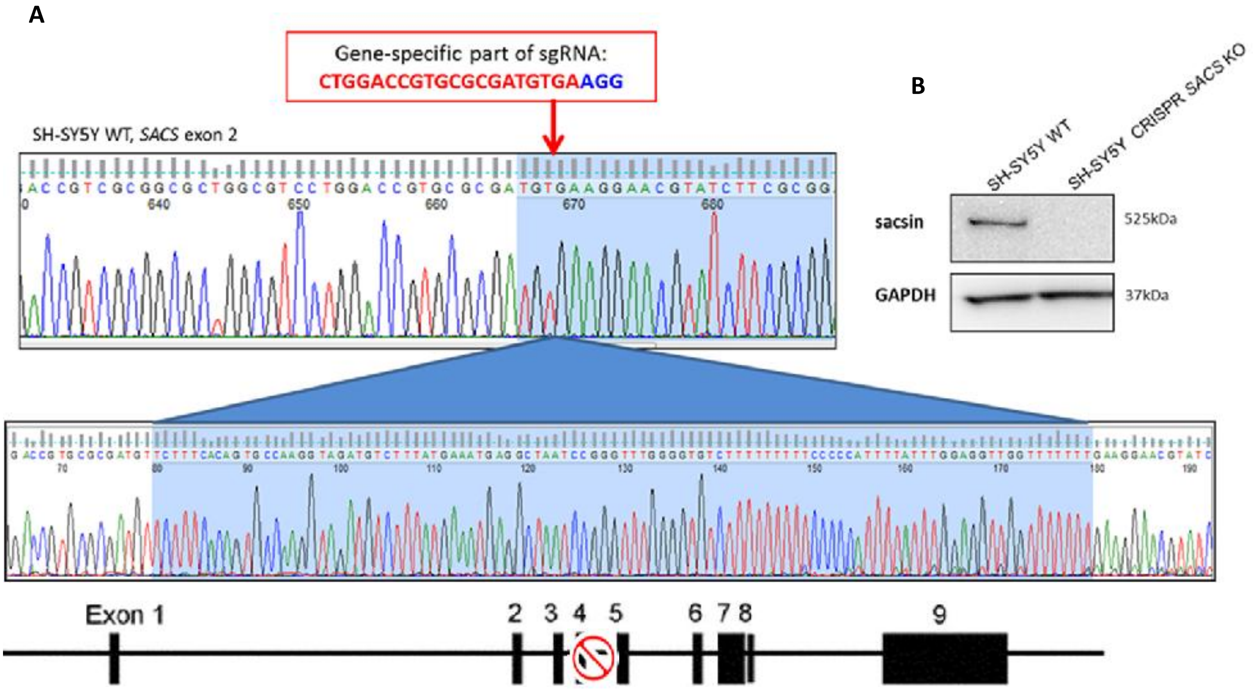

Supplementary Figure S1. Characterization of sacsin KO cell line generated by CRISPR/Cas9 gene editing technology. Genotype and protein expression of isolated clones from SH-SY5Y edited cells were verified by standard sequencing methods and Western blotting analyses. As shown in the figure the functional sgRNA was localized in exon 2 of the *SACS* gene. (**A**) Electropherogram analysis of the (**B**) The SH-SY5Y KO clone was characterized by a 100 bp insertion, resulting in a stop codon in exon 4. Sacsin protein levels, measured by Western blotting, were not detectable in KO cell line.

A

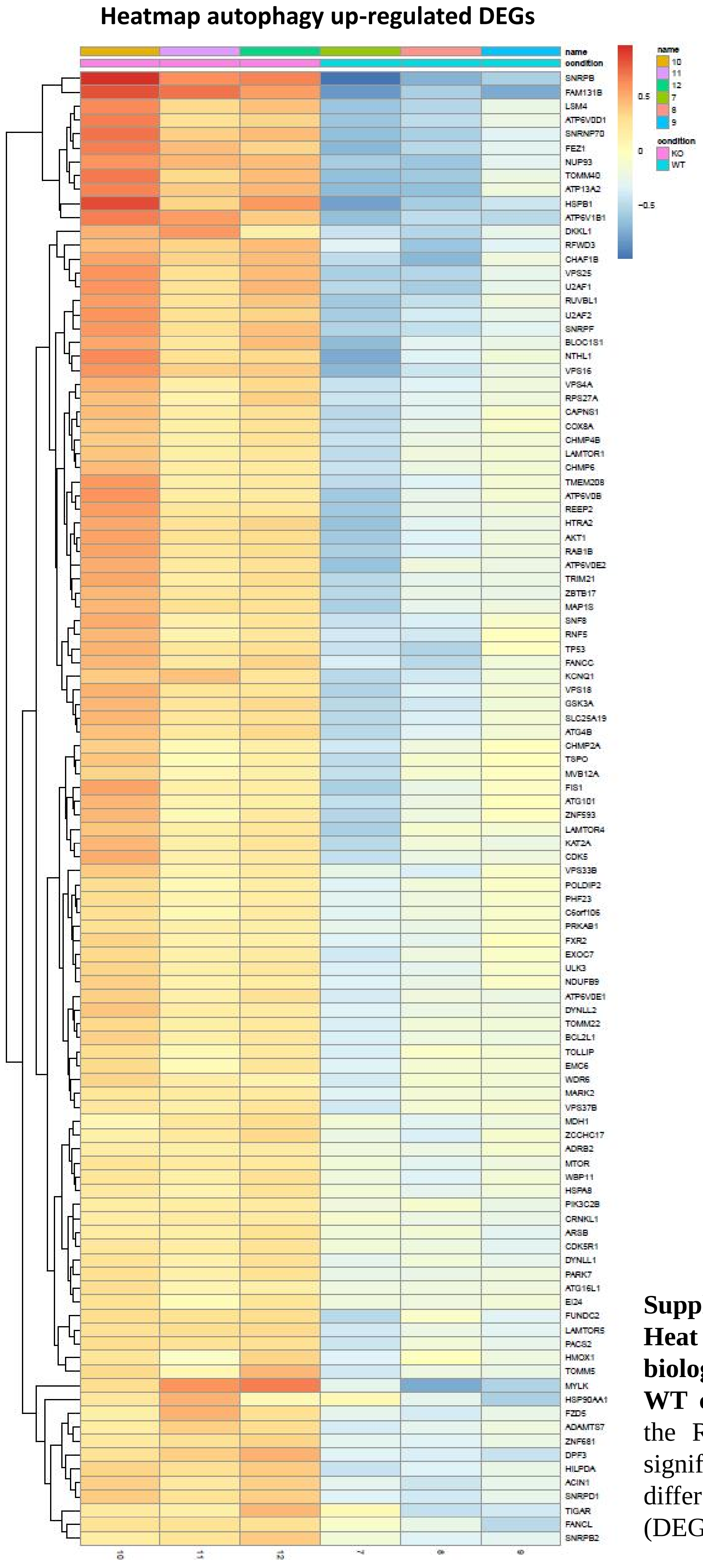

Supplementary Figure S2A. Heat map of autophagic biological process in KO vs WT cells. The figure shows the RNA-seq heat map for significantly up-regulated differentially expressed genes (DEGs).

B

Heatmap autophagy down-regulated DEGs

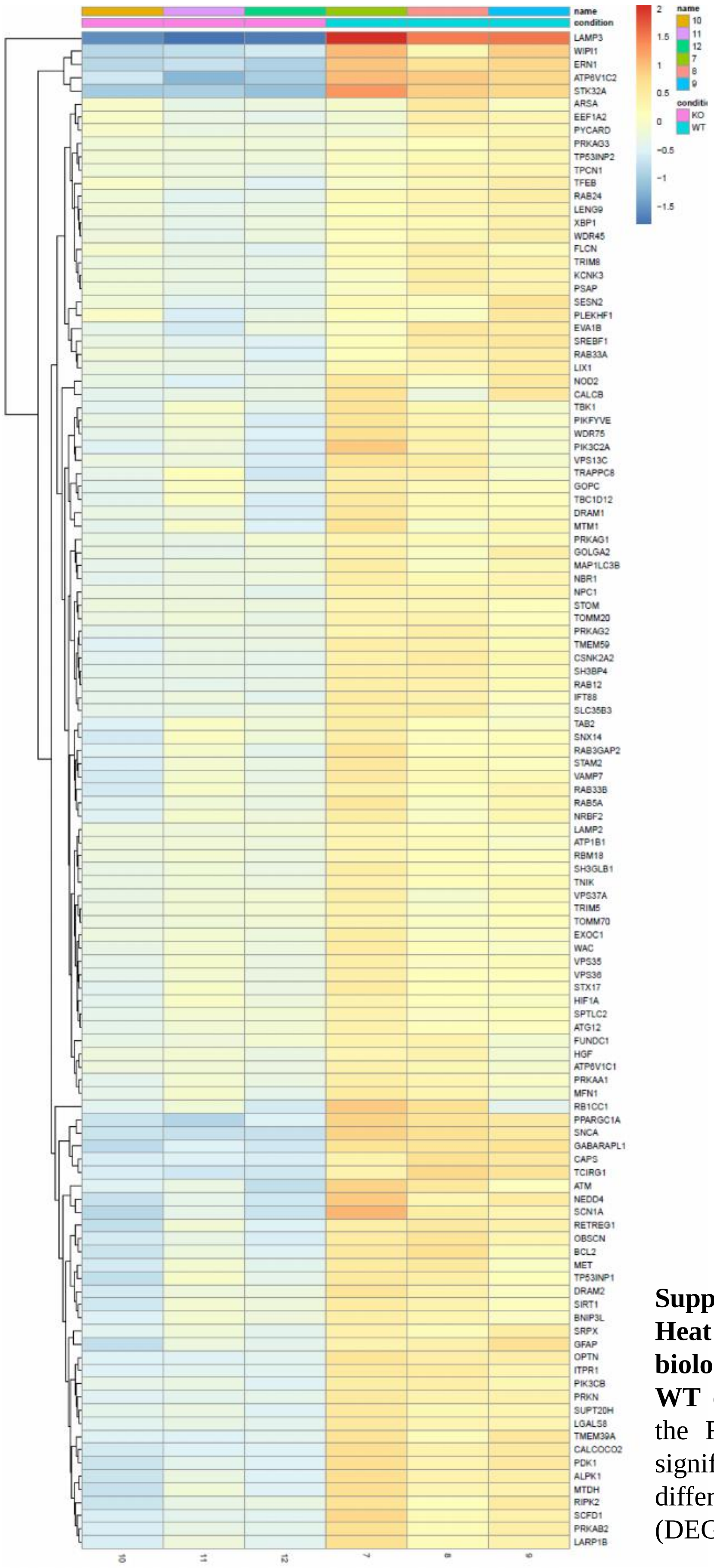

Supplementary Figure S2B. Heat map of autophagic biological process in KO vs WT cells. The figure shows the RNA-seq heat map for significantly down-regulated differentially expressed genes (DEGs).

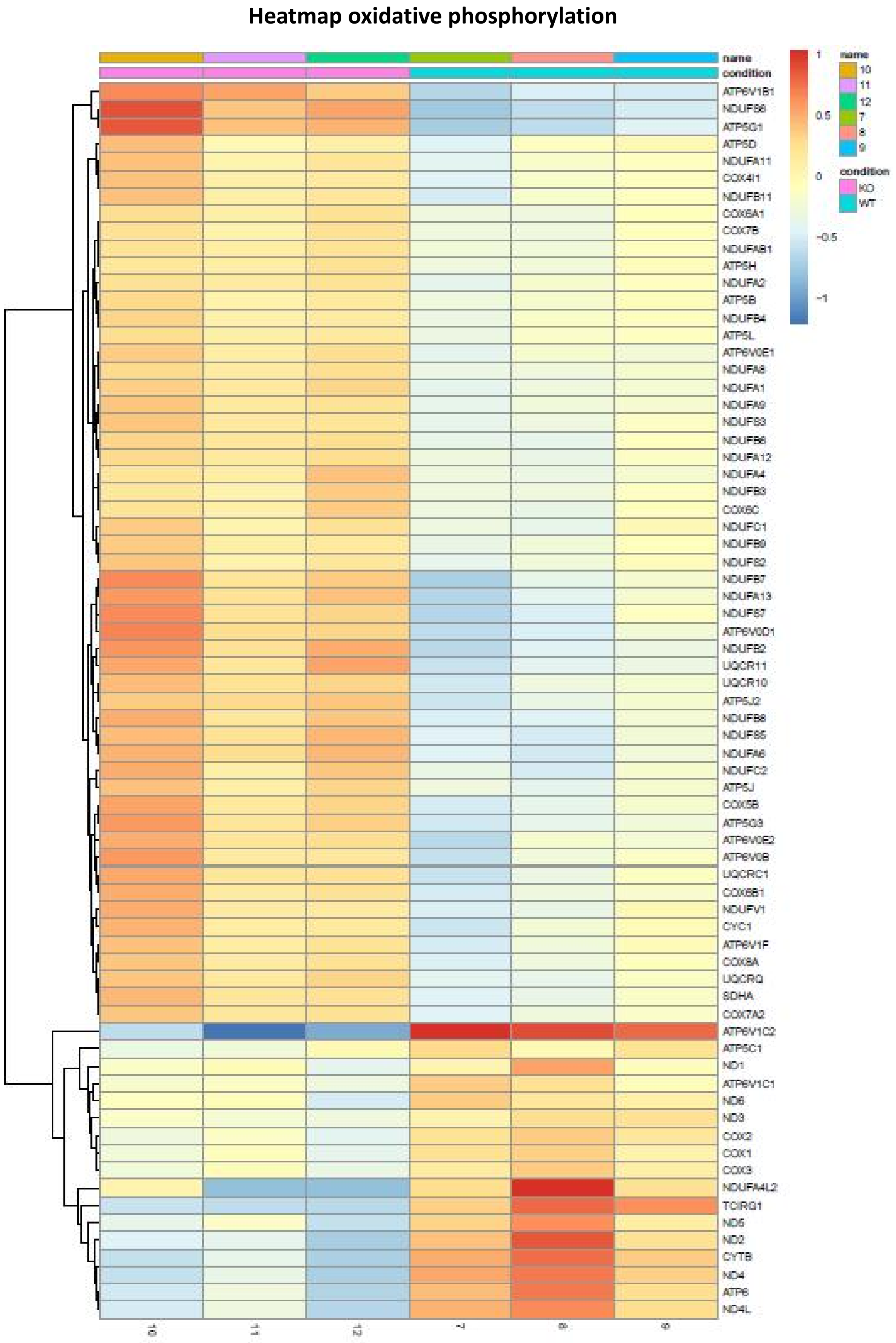

Supplementary Figure S3. Heat map of oxidative phosphorylation process in KO vs WT cells. The figure shows the RNA-seq heat map for oxidative metabolism genes.

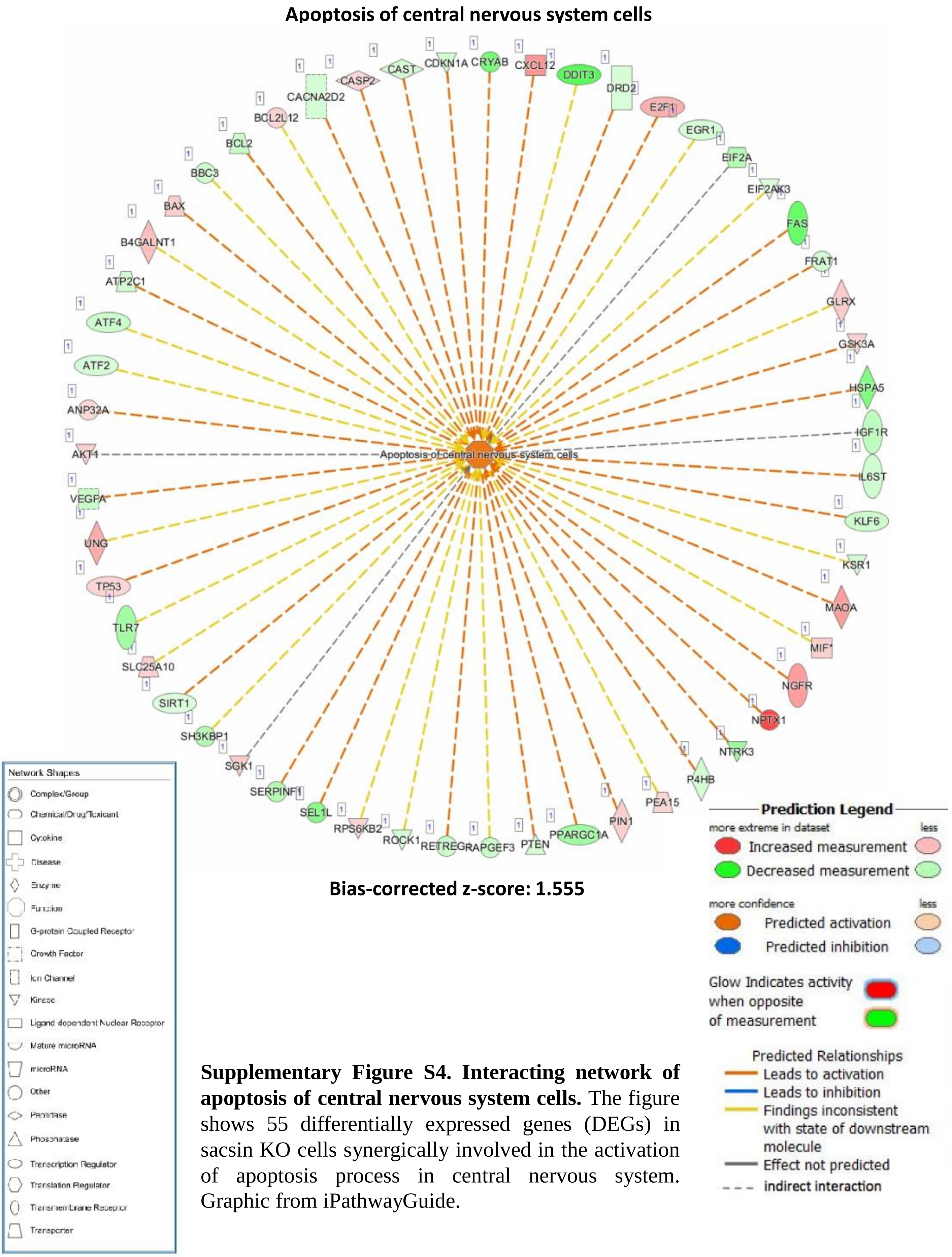

Supplementary Figure S4. Interacting network of apoptosis of central nervous system cells. The figure shows 55 differentially expressed genes (DEGs) in sacsin KO cells synergically involved in the activation of apoptosis process in central nervous system. Graphic from iPathwayGuide.
